# Dual-circulation: influence mechanism of ETS's carbon reduction and its spatiotemporal characteristics based on intensity modified SDID model

**DOI:** 10.1038/s41598-024-64250-x

**Published:** 2024-06-17

**Authors:** Xinmeng Tang, Tao Qin, Xin He, Moustafa Mohamed Nazief Haggag Kotb Kholaif

**Affiliations:** 1 School of Economics and Management, Beijing Forestry University, Haidian District, 35 Qinghua East Road, Beijing, 100091 China; 2 Post-Doctoral Research Workstation of Bank of Beijing, Beijing, 100033 China; 3 Accounting Department, Faculty of Commerce, Tanta University, Tanta, 31521 EL Gharbia Governorate Egypt

**Keywords:** Emission trading scheme, Carbon reduction, Policy intensity, Spatiotemporal characteristics, Climate sciences, Ecology, Environmental sciences

## Abstract

Traditional DID models overlook variations in policy intensity, causing estimation deviations from the actual situation and a limited understanding of the influence mechanism. In response, the Intensity Modified SDID Model is built to examine the influence mechanism of ETS's carbon reductions. Moreover, through model extensions, the study explores the spatiotemporal characteristics and heterogeneities of ETS’s effects. Results show that: (1) "Dual-circulation" influence mechanism is confirmed, where ETS directly contributes to carbon reductions (2.70% to 10.0% impact) through external pathways, and internal pathways continuously strengthen reduction effects, comprehensive mechanisms are thereby formed and enhanced based on interaction among internal and external pathways. (2) Reasonable ETS levels are estimated and proposed to achieve "Dual Carbon Target", constraining nationwide carbon quotas by 20 billion tons/year, increasing carbon trading volumes by 80 thousand tons/year, and elevating the carbon trading prices by 100 RMB (14 USD) per ton. (3) ETS's carbon reduction effects are identified with temporal and spatial characteristics, temporally, effects peak in the 4th period (*Event*_+*4*_) but diminish in the 5th period (*Event*_+*5*_), spatially, effects peak in areas distancing around 1000 km but disappear beyond 1500 km. (4) ETS also has synergistic effects with atmospheric pollution reduction, including industrial emissions of sulfur dioxide and smoke (dust), but are insignificant to industrial emissions of wastewater and solid waste.

## Introduction

The environmental and climate crises are widely recognized as paramount challenges facing humanity in the twenty-first century. Global warming and a myriad of ecological issues pose significant risks to both socioeconomic progress and physical well-being^1^. Consequently, the concept of sustainability often denoted as "green", has gained prominence in the twenty-first century. In response to these challenges, a multitude of concepts, policies, and strategies have surfaced. Notably, among these initiatives, the Emission Trading Scheme (ETS) stands out as a highly regarded tool for addressing environmental shifts, employing market mechanisms and pricing strategies.

ETS is designed to curb carbon emissions through a quota-based approach^2^. The process involves setting an emissions cap and distributing it to carbon market players^3^. Participants can benefit economically by trading any surplus quotas gained through production efficiency, providing an incentive for ETS participants to reduce emissions. Launched in 2013, China's ETS began as a pilot program in seven regions, these areas, representing around 20% of China's social wealth, labor, and energy consumption, demonstrated significant potential^2^. China's ETS is promising for the future in addition to having a sizable current volume.

An essential resource for decision-makers is the evaluation of the impacts of carbon reduction in the ETS pilot market. Given the exogenous nature of the ETS market, the model of Difference-in-Difference (DID) is predominantly utilized. DID's core idea is calculating the effectiveness of policies by contrasting their pre- and post-implementation variations between the control and treatment groups. Numerous studies, including those by studies^2,4–6^, affirm ETS’s positive effects as confirmed by DID.

However, the widely adopted DID models suffer from a significant deficiency by not considering changes in policy intensity. Conventional DID models quantify the time before and after policy adoption using straightforward 0–1 variables^7^. While DID models offer an alternative for problems that do not capture changes in policy intensity, they expose shortcomings when dealing with drastic changes in policy intensity, as is the case with ETS. After the establishment of ETS markets, prices and scale levels have undergone dramatic shifts, leading to a widening gap among pilot markets^8^. Consequently, traditional DID models, unable to adapt to variations in policy intensity, fail to accurately reflect the dynamic nature of the ETS market. This results in substantial estimation bias, hindering the precise evaluation of ETS's emission reduction effects and impeding a comprehensive understanding of its carbon reduction theoretical mechanism.

Neglecting policy intensity creates another significant gap in the existing literature concerning the influence mechanisms of ETS on carbon reductions. Despite the three main elements within ETS markets—carbon quotas, trading volumes, and prices—the specific interactions among these elements to form comprehensive mechanisms remain largely unexplored. The diverse elements within the ETS markets, including carbon quotas, drive increased trading volumes and subsequent rises in carbon trading prices^9,10^. While traditional DID models effectively capture the carbon emission intensity gap resulting from exogenous policy variables, there is a noticeable lack of research elucidating how internal diverse elements collaborate to establish an overarching mechanism for carbon emission reduction, indicating a current research gap.

Additionally, due to the limitations of policy intensity’ ignored by traditional DID models, the existing literature fails to accurately depict the spatiotemporal variations in ETS’s carbon reduction effects. In terms of temporal dynamics, the evolving trajectory of ETS carbon emission reduction efficiency over time remains elusive. Typically, there exists a time lag between policy implementation and effectiveness^7,11^. However, there are still questions about when the ETS carbon emission reduction effect begins to manifest itself and when it reaches its full impact because the standard DID model ignores policy intensity restrictions.

In the spatial aspect, the evolving trend of geographical variations in ETS’s carbon reduction effects eludes current understanding. Presently, the ETS market is confined to pilot provinces and cities, however, as per the spatial dependence theory, their carbon emission reduction effects exhibit spatial spillover effects^12,13^. There are still unanswered questions about the mechanics of the geographic spillover impact of the ETS's reduction in carbon emissions, including how it changes, the location of the attenuation boundary, and the identification of peak areas.

Addressing the identified research gaps, this study employs empirical data to examine how the ETS affects carbon reductions. The research follows a structured approach encompassing several key steps. Firstly, a novel Intensity Modified Spatial Difference-in-Difference (SDID) Model is developed, incorporating policy intensity as an indicator variable into the traditional SDID model. Secondly, leveraging the established Intensity Modified SDID Model, this study investigates the ETS's carbon reduction process, characterized by "dual-circulation". Thirdly, through extensions of the Intensity Modified SDID Model, incorporating dynamic effects and geographical attenuation processes, the research examines the spatiotemporal characteristics of the influence mechanism. Lastly, the paper investigates the heterogeneity of ETS carbon emission reduction effects.

**Figure 1:**
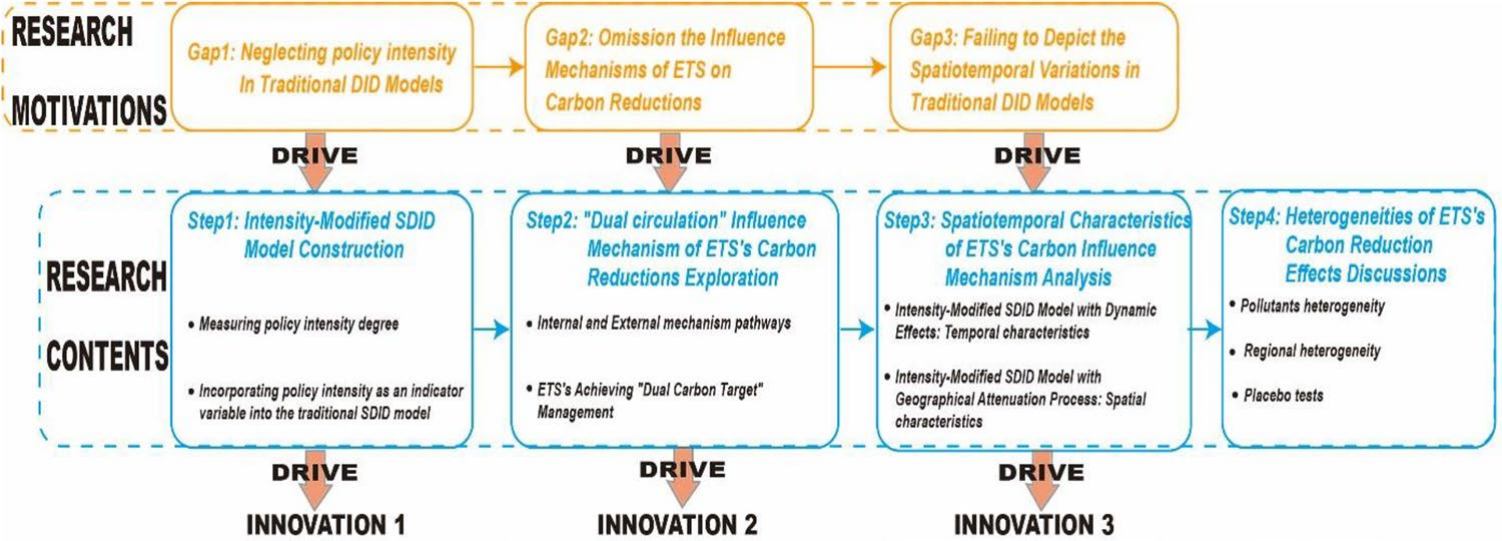
Research flow chart.

The organization of this study is as follows. Section "Theoretical analysis" provides the theoretical analysis of the "dual-circulation" influence mechanisms of ETS's carbon reduction. Further, in Section "Sample, variables, and methods", this study details the research sample, variables selection, and model construction. The empirical results of the ETS’ carbon reduction mechanism are presented in Section "Empirical results". Spatiotemporal characteristics of ETS's carbon reduction influence mechanism are discussed in Section "Discussions". Heterogeneities of ETS's carbon reduction effects are explored in Section "Research conclusions and implications". This study summarizes the research conclusions and practical implications in Sect. 7. Figure 1 provides a summary of the fundamental research framework.

## Theoretical analysis

**Figure 2:**
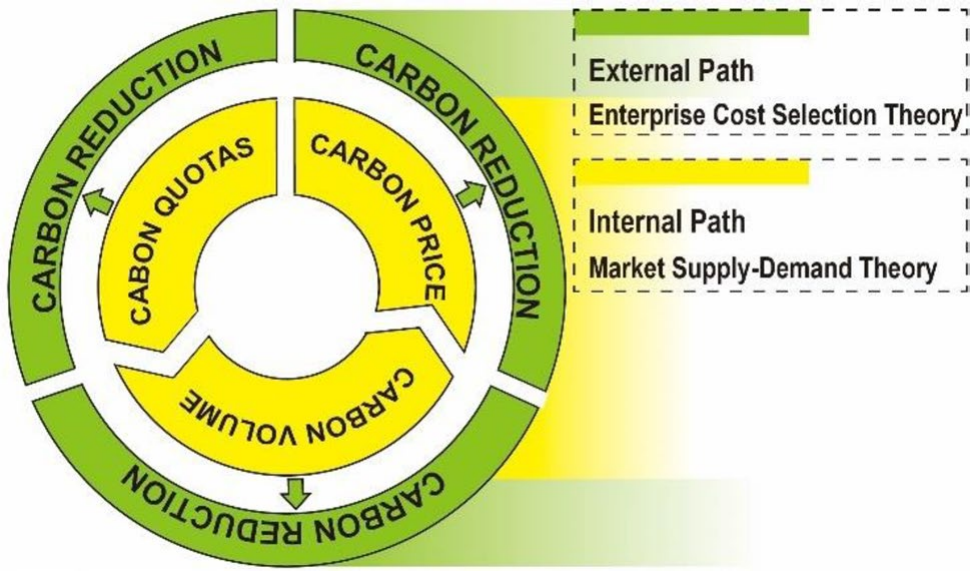
ETS's influence mechanism on carbon reduction.

"Dual circulation" is the principal characteristic delineated within the influence mechanism of ETS's carbon reduction process. The internal and external mechanism pathways overlap and converge to form a comprehensive ETS carbon reduction influence mechanism process, as shown in Fig. 2.

The essential theory underpinning the external mechanism pathway is rooted in the theory of enterprise cost selection^14^. Confronted with emissions quotas, companies typically consider two primary strategies: upgrading equipment to curtail emissions or compensating for excess emissions by acquiring carbon credits through market transactions^15^. The transaction costs of the carbon market incentivize companies to pursue carbon reduction strategies. Moreover, the contracting of carbon quotas and the escalation in trading volume contribute to heightened carbon prices, which will push up the cost of purchasing carbon volumes to offset excess emissions, thus, companies' motivations to reduce carbon emissions increase^16^.

For carbon quotas, implementing the carbon quota management system has transformed carbon emissions from arbitrary and inconsequential entities into commodities^17^. This alteration introduces an additional cost for enterprises' exceeding emissions, compelling them to invest in advancing green technologies for carbon emission reduction or carbon efficiency improvement^16^. For carbon trading volumes, carbon quotas encourage over-emission companies to participate in the ETS market, thereby fostering increased activity in carbon trading. It indicates that restricted companies are seeking solutions to excessive emissions. Beyond mere participation in market transactions, companies are prompted to organically diminish carbon emissions or explore avenues for enhancing carbon efficiency^15,16^. For carbon trading prices, elevated carbon prices translate into heightened costs for companies grappling with excessive emissions, forcing companies to explore remedies outside the ETS market, such as replacing production equipment^10^.

Market supply–demand theory underpins the internal mechanism pathway. Introducing carbon emission quotas incentivizes enterprises to engage in carbon market trading, thereby augmenting market liquidity and bolstering trading volume^16^. Consistent with supply–demand theory, in a scenario of stable carbon supply, the heightened demand serves as a driving force, further propelling carbon prices upwards.

Carbon quotas, carbon trading volume, and carbon trading prices exist in a symbiotic and collaborative relationship within the framework of ETS. Functioning as a mandatory environmental policy, the efficacy of ETS initiates with compulsory constraints imposed by governments, compelling companies to seek resolutions, either through equipment upgrades or carbon purchasing to offset emissions^14^. Consequently, it motivates companies to engage actively in the ETS market, thereby augmenting both market liquidity and trading volumes^16^. According to supply and demand theory, the stable carbon supply situation propels carbon trading prices upwards^8^. In essence, the limitations imposed by carbon quotas amplify demand within ETS markets, consequently driving an increase in carbon prices.

In summation, the integration of the internal and external pathways yields the carbon reduction effect mechanism, constituting the "dual circulation" feature. The external mechanism path serves as the foundation, with the three components assuming direct roles in effecting carbon reduction. Simultaneously, the internal mechanism path enhances this effect through the interrelation and collaboration of three elements, operating synergistically with the external path to achieve a heightened reduction effect.

## Sample, variables, and methods

### Research sample

This study adopts panel data, including cross-section and time dimensions. For the cross-section aspect, since China's carbon trading pilot areas are basically established at the provincial and municipal levels, the sample of this study spans all provinces and municipalities in China, except for Taiwan, Hong Kong, Macau, and Tibet, where data are not available, Also, for the period aspect, considering that China's carbon trading pilot areas were established between 2013 and 2016, the time span is from 2010 to 2019.

### Variables constructions

#### Carbon emissions (CB)

This study captures both intensity and efficiency aspects, which could provide robust evidence for each other.

1. Carbon emission intensity (CBEM)

The outcome variables refer to the intensity of carbon emission. Consistent with existing literature^10,18^, data on carbon emission is adopted from China Emission Accounts and Datasets (CEADs). it is anticipated that using a panel at the province level would more accurately measure the extent of decrease resulting from the implementation of the ETS, as the pilot unit is primarily established in provinces and municipalities.

2. Carbon emission efficiency (CBEF)

Furthermore, since carbon emission is generally considered a consequence of human subsistence activities (Hu et al., 2023; Shafik, 1994), single carbon output proxy (i.e., carbon emissions) that do not consider the environmental inputs are narrow and limited. The carbon efficiency indicators reflecting input and output thereby become another suitable outcome variable. Specifically, following studies^13,19^, the Slack-Based Measure Data Envelopment Analysis (SSBM-DEA) method is adopted to calculate carbon efficiency.

Moreover, to meet the calculation requirements of the SSBM-DEA method, input indexes (physical capital stock, population, energy consumption), expected output index (real GDP), and unexpected output indexes (carbon dioxide emission, industrial sulfur dioxide emission, and industrial wasted water emission) are added into SSBM-DEA model.

#### Policy measurement

**Figure 3:**
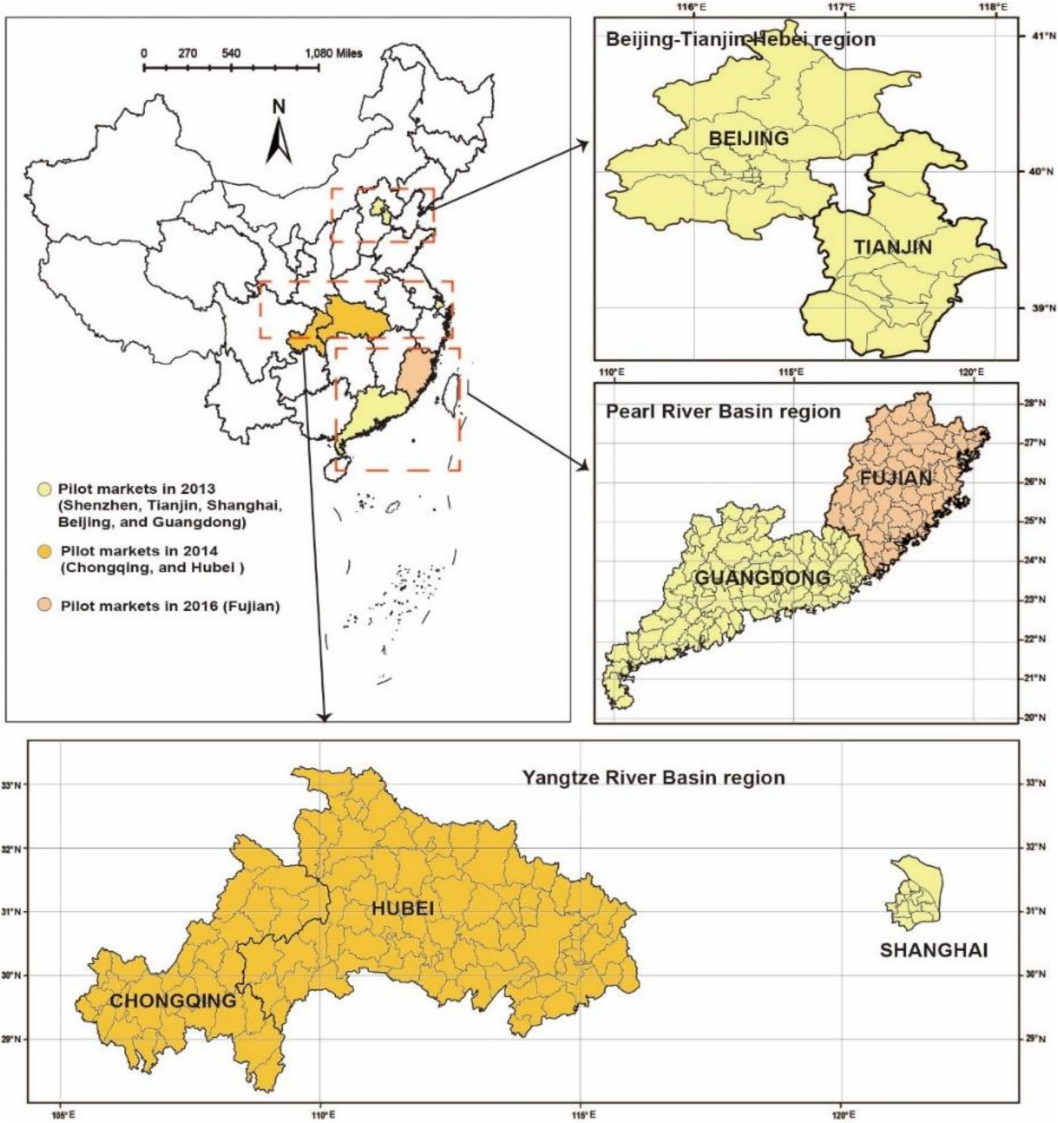
ETS policy implementation process. Basemap data from the Resource and Environment Science and Data Center, Institute of Geographic Sciences and Natural Resources Research, Chinese Academy of Sciences (https://doi.org/10.12078/2023010103); Software version is ArcMap 10.6, URL is from https://www.esri.com/en-us/arcgis/products/indext.

The research subject in this article is ETS, a policy that has undergone successive implementation in eight pilot areas. To be more precise, carbon markets were formed in Shenzhen, Tianjin, Shanghai, Beijing, and Guangdong provinces in 2013, followed by Chongqing and Hubei in 2014. Finally, Fujian became the last pilot area to develop a carbon market in 2016, as depicted in Fig. 3.

Furthermore, in light of the research focus on the inherent influence mechanism of the ETS, as well as the necessity of considering policy intensity in traditional SDID models, this article places particular emphasis on the three essential elements of ETS, carbon quotas, carbon trading price volume, carbon trading price. These elements are also integrated into the study as indicators of policy intensity.

#### Heterogenous pollutants variables

In the examination of pollutant heterogeneity, more pollutants are included. The focus is directed towards industrial enterprises as the primary sources of pollution, with particular attention given to various industrial pollutants. This encompasses industrial emissions such as sulfur dioxide, smoke (dust), solid waste, and wastewater.

#### Control variables

The IPAT and STIRPAT models are widely acknowledged formulas used to analyze the environmental impacts of human activities. Three factors proposed in the IPAT and STIRPAT models are widely used in empirical research^20,21^. Following this notion, control variables are derived from three perspectives as outlined below.

1. Affluence factors

Gross domestic product (*GDP*). GDP is the most direct reflection and representative of national wealth, so this article uses GDP as the control variable. Moreover, an inflation-adjusted GDP index with 2010 as the base year is used to deflate price fluctuations and counteract inflation's potential impact over time.

Trade openness (*TO*). The total trade volume in GDP is adopted as a control variable to reflect trade openness in this study. Trade volume reflects affluence factors in terms of the international trading market. Furthermore, trade volume is denominated in RMB.

Environmental tax (*ET*). The level of environmental tax reflects the scale of highly polluting industries, it also reflects the government's regulatory efforts. Consistent with existing literature^22^, environmental tax is included as a control variable in the STIRPAT model.

Traffic investment (*TI*). The academic community increasingly believes that infrastructure investment, especially transportation, reflects the adequacy of national wealth^23^. Also, the transportation construction industry usually belongs to secondary industries with high pollutant emissions, so this article uses transportation investment as the control variable.

(2) Population factors

Employment (*EM*). Environmental quality refelts the consequences of human activities. Therefore, employment rather than population is a suitable index for controlling environmental impacts. The total amount of employment is adopted in this article as a control variable.

(3) Technology factors

Energy consumption (*EC*). In traditional STIRPAT, technology is mainly driven by energy consumption^24,25^, leading to alterations in the environment. Consistent with this, this article uses total energy consumption as the control variable.

Renewable energy development (*RE*). Nations are progressively embracing novel forms of renewable energy to supplant conventional energy sources as a catalyst for technological advancement^26^, resulting in greener environmental consequences. In STIRPAT, the incorporation of novel renewable energy sources is considered as a controlling variable for environmental quality.

R&D investment (*RD*). Another important driving force for technological progress lies in investment, especially in research and development. The current understanding of the environmental impacts of R&D expenditure remains uncertain but aligns with previous research findings^27^, R&D investment is included in STIRPAT as a control factor for environmental quality.

Patent application (*PA*). The quantifiable and immediate result of technological advancement is the quantity of patent applications, which serves as the benchmark for translating technological accomplishments. Consistent with existing literature^28^, the total amount of patent applications is included in STIRPAT as a control factor for environmental quality.

The factors utilized in this investigation are consolidated in Table 1.

**Table 1:** Summary of variables.

	Variable	Abb	Data resource
Outcome variables	Carbon emission intensity	*CBEM*	Data are from China Emission Accounts and Datasets (CEADs, https://www.ceads.net/)
	Carbon emission efficiency	*CBEF*	Calculated by this study, data are from the CEADs and Wind Database
Policy variables	Policy implementation	*POST* × *TREND*	Calculated by this study
	Carbon trading prices	*Price*	Data are from Wind Database
	Carbon trading volume s	*Volume*	Data are from the Wind Database
	Carbon quotas	*Quota*	Data are from government/municipality reports
Other pollutants variables	Industrial sulfur dioxide emission	*ISDE*	Data are from China Environmental Statistical Yearbook (Versions of 2011 to 2020)
	Industrial smoke (dust) emission	*ISE*	Data are from China Environmental Statistical Yearbook (Versions of 2011 to 2020)
	Industrial wastewater emission	*IWWE*	Data are from China Environmental Statistical Yearbook (Versions of 2011 to 2020)
	Industrial solid waste emission	*ISWE*	Data are from China Environmental Statistical Yearbook (Versions of 2011 to 2020)
Control variables	Gross domestic product	*GDP*	Data are from the WIND Database
	Trade openness	*TO*	Data are from the WIND Database
	Environmental tax	*ET*	Data are from China Environmental Statistical Yearbook (Versions of 2011 to 2020)
	Traffic investment	*TI*	Data are from the WIND Database
	Employment	*EM*	Data are from the WIND Database
	Energy consumption	*EC*	Data are from the China Energy Statistical Yearbook (Versions of 2011 to 2020)
	Renewable energy development	*RE*	Data are from the China Energy Statistical Yearbook (Versions of 2011 to 2020)
	R&D investment	*RD*	Data are from the WIND Database
	Patent application	*PA*	Data are from the WIND Database

### Construction process of the Intensity Modified SDID Model

#### Significance of Intensity Modified SDID Model

**Figure 4:**
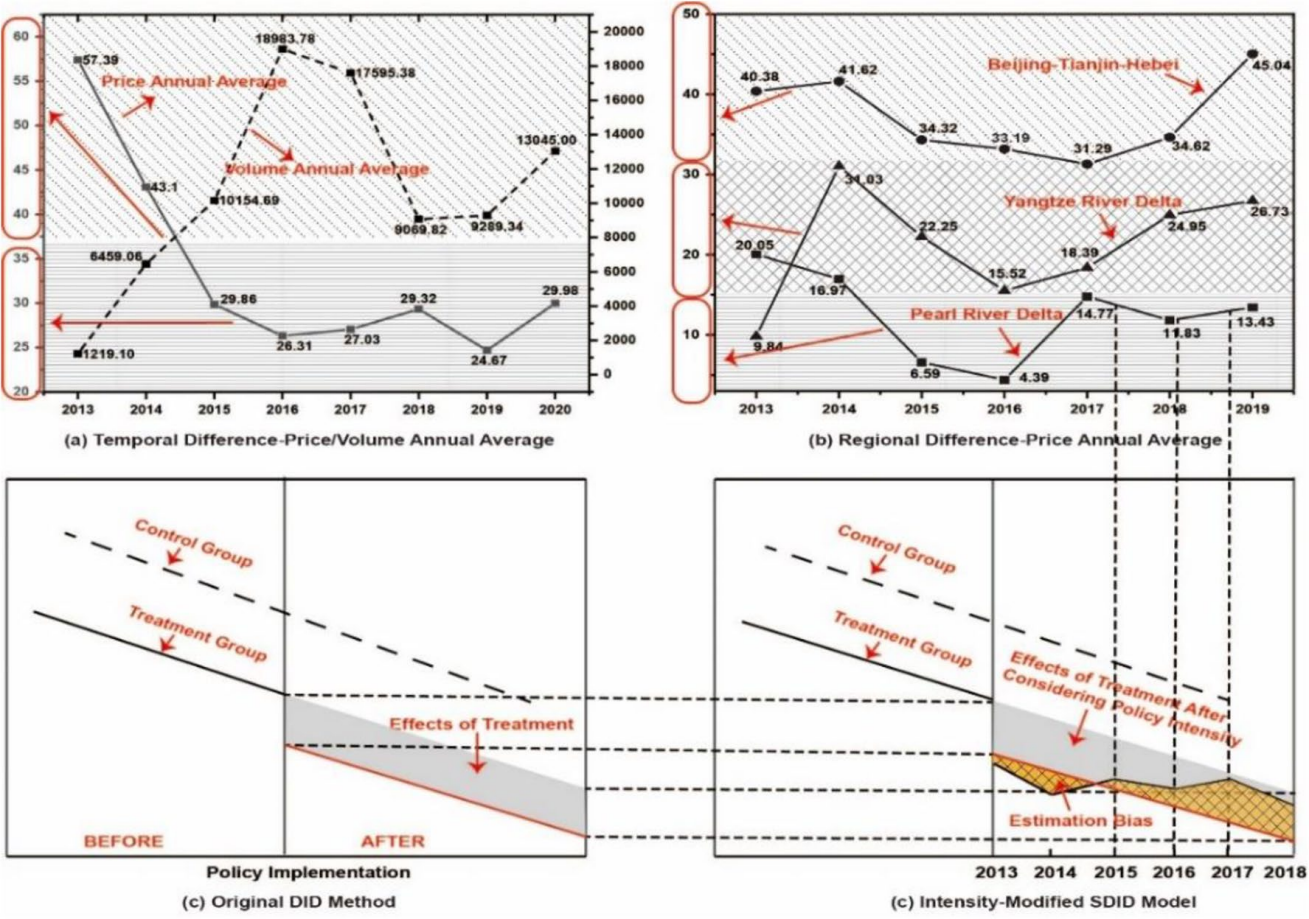
Significance of Intensity Modified SDID Model.

Empirical evidence demonstrates substantial variations in the intensity of ETS, both temporally and regionally. Neglecting to account for policy intensity introduces notable model biases. From temporal standpoints, as illustrated in Fig. 4(a), significant discrepancies exist in the yearly mean fluctuations of carbon trading prices and volumes. More precisely, the yearly mean of carbon trading prices is only 50% of the beginning level, whilst the yearly mean of carbon trading volumes undergoes a tenfold rise. Also, a substantial gap is evident among pilot markets when examining regional intensity differentials, as depicted in Fig. 4(b). The yearly mean carbon trading price in the Beijing-Tianjin-Hebei region, which has the highest policy intensity, is nearly three times higher than the region with the lowest policy intensity. These underscore the significant variations in policy intensity since the inception of the ETS, emphasizing that the omission of policy intensity considerations results in model bias.

Nevertheless, a notable limitation of traditional DID is the inability to capture variations in policy intensity and to reflect disparities in policy effectiveness between treatment and control groups. As depicted in Fig. 4(c), conventional DID models dichotomize policy into a binary 0–1 variable before and after treatment, neglecting its continuous nature when evaluating the impact of the average treatment policy^7^. In contrast, Fig. 4(d) illustrates the newly formulated Policy-Intensity Modified SDID Model in this paper to address the shortcomings of traditional DID models. In real-world scenarios, policy intensity undergoes real-time changes. Consistent use of traditional DID models would result in estimation bias (depicted in the orange part of Fig. 4 (d)), contradicting the actual situation and failing to accurately capture the efficacy of policy implementation.

**Figure 5:**
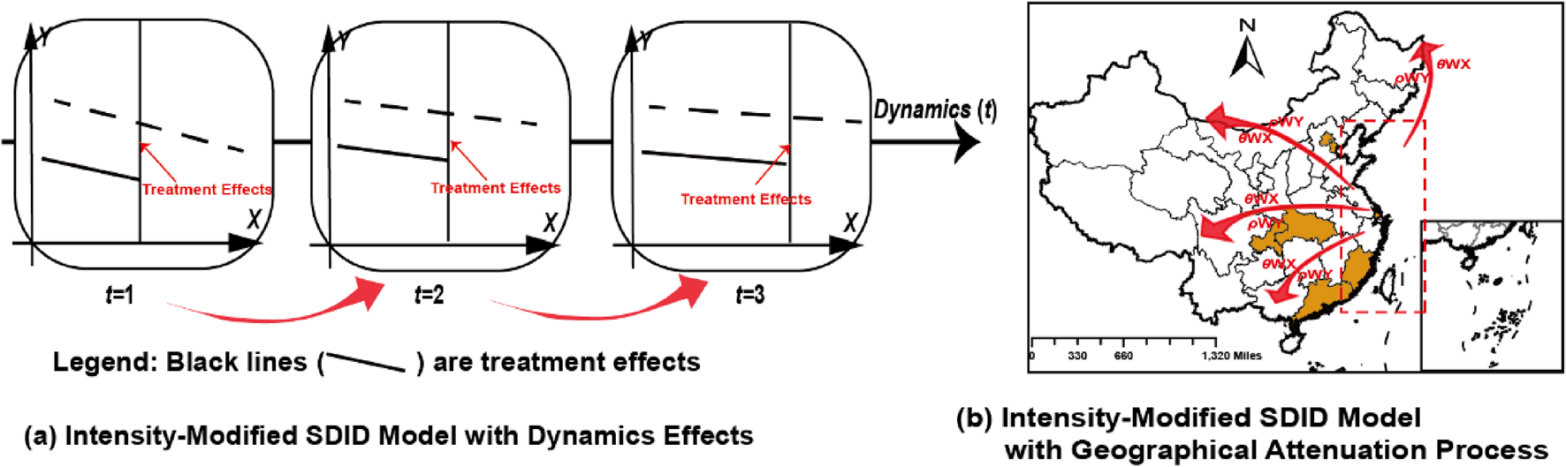
Extensions of Intensity Modified SDID Model. Basemap data from the Resource and Environment Science and Data Center, Institute of Geographic Sciences and Natural Resources Research, Chinese Academy of Sciences (https://doi.org/10.12078/2023010103); Software version is ArcMap 10.6, URL is from https://www.esri.com/en-us/arcgis/products/indext.

By incorporating policy intensity into SDID models, another two significant deficiencies in existing DID models could be addressed through the extensions to the Intensity Modified SDID Model. Firstly, the limitation of neglecting changes in treatment effects over time is addressed by constructing the Intensity Modified SDID Model with Dynamics Effects. Traditional DID models solely compare treatment effects prior to and following policy adoption, overlooking the temporal evolution of policy intensity^7,11^. As illustrated in Fig. 5(a), the capture of policy intensity within SDID models enables the observation of dynamic changes in treatment effects (i.e., temporal characteristics) through the construction of the Intensity Modified SDID Model with Dynamics Effects.

Secondly, the limitation of overlooking the geographical attenuation process of treatment effects is mitigated by constructing the Intensity Modified SDID Model with Geographical Attenuation Process. Drawing from the theory of spatial dependence, treatment effects diminish with increasing geographical distance, as recognized in geographical attenuation laws^12,13^, which is often neglected in traditional DID models. As depicted in Fig. 5(b), the incorporation of policy intensity within SDID models facilitates the observation of geographical attenuation processes (i.e., spatial characteristics) through the construction of the Intensity Modified SDID Model with Geographical Attenuation Process.

#### Baseline models

Building upon the STIRPAT model^13^, the baseline model incorporates the explanatory components related to ETS and carbon reduction, as depicted in Eq. (1).

1 $$ \begin{gathered} CBEM_{i,t} = \alpha_{0} + \alpha_{1} POLICY_{i,t} + \alpha_{2} GDP_{i,t} + \alpha_{3} TO_{i,t} + \alpha_{4} ET_{i,t} + \alpha_{5} TI_{i,t} + \alpha_{6} EM_{i,t} \hfill \\ \, + \alpha_{7} EC_{i,t} + \alpha_{8} RE_{i,t} + \alpha_{9} RD_{i,t} + \alpha_{10} PA_{i,t} + \varepsilon_{i,t} ,\varepsilon_{i,t} \sim N(0,\sigma_{i,t}^{2} ), \hfill \\ \end{gathered} $$

where *CB*_*i,t*_ denotes the explained variable, including the intensity and efficiency of carbon emission at year *t* of province* i (CBEM*_*i,t*_* and CBEF*_*i,t*_*)*; *POLICY*_*i,t*_ denotes the policy variable at year *t* of province *i*; Furthermore, *GDP*_*i,t*_, *TO*_*i,t*_, *ET*_*i,t*_, *TI*_*i,t*_, *EM*_*i,t*_, *EC*_*i,t*_, *RE*_*i,t*_, *RD*_*i,t*_, and *PA*_*i,t*_ stand for the control variables at year *t* of province* i*; the variable *ε* encompasses random errors.

#### SDID models

The policy subject in this article is ETS, which is a standard quasi-natural experiment. The micro-polluting enterprises examined in this article are unable to intervene or affect the implementation of this national law, that is, for micro-enterprises, the implementation of ETS is an exogenous event^29^. Therefore, Consequently, the DID model, a popular tool for measuring the success of policies^10^, is introduced in Eq. (2).

2 $$ \begin{gathered} CB_{i,t} = \beta_{0} + \beta_{1} TREND_{i,i} \times POST_{i,i} + \beta_{2} GDP_{i,t} + \beta_{3} TO_{i,t} + \beta_{4} ET_{i,t} + \beta_{5} TI_{i,t} + \beta_{6} EM_{i,t} \hfill \\ \, + \beta_{7} EC_{i,t} + \beta_{8} RE_{i,t} + \beta_{9} RD_{i,t} + \beta_{10} PA_{i,t} + \varepsilon_{i,t} ,\varepsilon_{i,t} \sim N(0,\sigma_{i,t}^{2} ), \hfill \\ \end{gathered} $$

where the variable of *TREND*_*i,t*_ denotes a dummy variable to capture the treatment group, it equals 1 when province *i* is selected as the ETS pilot region, it equals 0 otherwise; the variable of *POST*_*i,t*_ denotes a dummy variable to capture time difference, being equal to 1 in cases when ETS regions begin in year t and subsequent years, and 0 in all other cases.

Based on the theory of spatial dependence on environmental quality, environmental quality, especially pollutants with gas properties, such as carbon dioxide, is highly correlated over geographical distance. As the atmosphere flows, carbon dioxide flows in space^12,13^. Therefore, the traditional DID models are extended to the SDID models^7^, which consider the spatial factor, as shown in Eq. (3).

3 $$ \begin{gathered} CB_{i,t} = \gamma_{0} + \rho \sum\limits_{j = 1}^{N} {W_{i,t} CBEM_{i,t} } + \gamma_{1} TREND_{i,i} \times POST_{i,i} + \gamma_{2} \sum\limits_{j = 1}^{N} {W_{i,t} } TREND_{i,i} \times POST_{i,i} \hfill \\ \, + \phi X_{i,t} + \theta \sum\limits_{j = 1}^{N} {W_{i,t} X_{i,t} } + c_{i} + u_{t} + \varepsilon_{i,t} , \, \varepsilon_{i,t} \sim N(0,\sigma_{i,t}^{2} ), \hfill \\ \end{gathered} $$

where *W*_*i,t*_ denotes the spatial weight matrix. When it comes to spatial dependence, carbon emissions have the clearest link between distance and amount, *W*_*i,t*_ is thereby directly constructed from the distance between provinces, as shown in Eq. (4). The matrix of control factors is captured by *X*; *c* stands for the individual effect and *u* for the time fixed effect.

4 $$ W_{i,j} = \left\{ {\begin{array}{*{20}c} {\frac{1}{{d_{i,j}^{2} }},i \ne j} \\ {0, \, i = j} \\ \end{array} } \right., $$

where *d*_*i,j*_ represents the geographical distance, measured in terms of longitude and latitude, between the capital cities of province *i* and *j*.

#### Intensity Modified SDID Model

As previously highlighted, the inherent limitation of traditional SDID models lies in their inability to account for variations in policy intensity, leading to an imprecise assessment of policy implementation effectiveness. In response, this study introduces the Intensity Modified SDID Model, an extension of conventional SDID models, specifically designed to address this deficiency.

*STEP 1: *Calculate the policy intensity as variable *I*, which is dynamic over time and signifies the fluctuations in policy intensity.

*STEP 2:* Incorporate policy intensity variable *I* into the traditional SDID model. The resulting model, termed the Intensity Modified SDID Model, can be derived using Eq. (5).

5 $$ \begin{gathered} CB_{i,t} = \gamma_{0} + \rho \sum\limits_{j = 1}^{N} {W_{i,t} CBEM_{i,t} } + \gamma_{1} TREND_{i,i} \times POST_{i,i} \times I + \gamma_{2} \sum\limits_{j = 1}^{N} {W_{i,t} } TREND_{i,i} \times POST_{i,i} \times I \hfill \\ \, + \phi X_{i,t} + \theta \sum\limits_{j = 1}^{N} {W_{i,t} X_{i,t} } + c_{i} + u_{t} + \varepsilon_{i,t} , \, \varepsilon_{i,t} \sim N(0,\sigma_{i,t}^{2} ), \hfill \\ \end{gathered} $$

In the practical context of this study, policy intensity encompasses three dimensions, carbon quotas, carbon trading price, and carbon trading volume. Consequently, Eq. (5) is extended to the following specific Eqs. (6) to (8).

6 $$ \begin{gathered} CB_{i,t} = \gamma_{0} + \rho \sum\limits_{j = 1}^{N} {W_{i,t} CBEM_{i,t} } + \gamma_{1} TREND_{i,i} \times POST_{i,i} \times I^{quota} + \gamma_{2} \sum\limits_{j = 1}^{N} {W_{i,t} } TREND_{i,i} \times POST_{i,i} \times I^{quota} \hfill \\ \, + \phi X_{i,t} + \theta \sum\limits_{j = 1}^{N} {W_{i,t} X_{i,t} } + c_{i} + u_{t} + \varepsilon_{i,t} , \, \varepsilon_{i,t} \sim N(0,\sigma_{i,t}^{2} ), \hfill \\ \end{gathered} $$

where *I*^*quota*^ denotes the proxy for policy intensity using carbon quotas.

7 $$ \begin{gathered} CB_{i,t} = \gamma_{0} + \rho \sum\limits_{j = 1}^{N} {W_{i,t} CBEM_{i,t} } + \gamma_{1} TREND_{i,i} \times POST_{i,i} \times I^{volume} + \gamma_{2} \sum\limits_{j = 1}^{N} {W_{i,t} } TREND_{i,i} \times POST_{i,i} \times I^{volume} \hfill \\ \, + \phi X_{i,t} + \theta \sum\limits_{j = 1}^{N} {W_{i,t} X_{i,t} } + c_{i} + u_{t} + \varepsilon_{i,t} , \, \varepsilon_{i,t} \sim N(0,\sigma_{i,t}^{2} ), \hfill \\ \end{gathered} $$

where *I*^*volume*^ denotes the proxy for policy intensity using carbon trading volumes.

8 $$ \begin{gathered} CBEM_{i,t} = \gamma_{0} + \rho \sum\limits_{j = 1}^{N} {W_{i,t} CBEM_{i,t} } + \gamma_{1} TREND_{i,i} \times POST_{i,i} \times I^{price} + \gamma_{2} \sum\limits_{j = 1}^{N} {W_{i,t} } TREND_{i,i} \times POST_{i,i} \times I^{price} \hfill \\ \, + \phi X_{i,t} + \theta \sum\limits_{j = 1}^{N} {W_{i,t} X_{i,t} } + c_{i} + u_{t} + \varepsilon_{i,t} , \, \varepsilon_{i,t} \sim N(0,\sigma_{i,t}^{2} ), \hfill \\ \end{gathered} $$

where* I*^*price*^ denotes the proxy for policy intensity using carbon trading prices.

#### Intensity Modified SDID Model with Dynamic Effects

To capture changes in ETS’s effects over time, the ESA method is introduced. The main objectives of ESA in the context of DID are twofold: firstly, to examine the validity of the parallel trend hypothesis in the DID approach, and secondly, to gain a more comprehensive understanding of the variations in treatment effects over time^11,30^.

This study incorporates time dummy variables of three pre-ETS stages and seven post-ETS phases, drawing on previous research^7^, which are *Event*_*-3*_, *Event*_*-2*_, …, *Event*_*0*_, …, *Event*_+*7*_. Unlike Eq. (5) captures the average treatment effect post-policy, *η* and *γ* in Eq. (9) specifically measure the treatment effect of the specific period.

9 $$ \begin{gathered} CB_{i,t} = \gamma_{0} + \rho \sum\limits_{j = 1}^{N} {W_{i,t} CBEM_{i,t} } + \sum\limits_{j = 1}^{N} {\sum\limits_{\iota = 1}^{m} {\left( {\eta_{ - \iota } + \gamma_{ - \iota } W_{i,t} } \right) \times Event_{ - \iota } \times I} } + \left( {\eta_{0} + \gamma_{0} W_{i,t} } \right) \times Event_{0} \times I \hfill \\ \, + \sum\limits_{j = 1}^{N} {\sum\limits_{\iota = 1}^{m} {\left( {\eta_{ + \iota } + \gamma_{ + \iota } W_{i,t} } \right) \times Event_{ + \iota } \times I} } + \phi X_{i,t} + \theta \sum\limits_{j = 1}^{N} {W_{i,t} X_{i,t} } + c_{i} + u_{t} + \varepsilon_{i,t} , \, \varepsilon_{i,t} \sim N(0,\sigma_{i,t}^{2} ), \hfill \\ \end{gathered} $$

#### Intensity Modified SDID Model with Geographical Attenuation Process

Threshold distance spatial weight matrices are created in order to record the geographical attenuation process. More precisely, referring to Eqs. (10) and (11), supposing that the maximum distance is denoted as [*d*_min_, *d*_max_], the incremental distance is represented by *γ*, and the distance threshold is denoted as *d*. The square of the reciprocal of the two provincial distances are elements of the matrix if *d*_*ij*_ > *d*, 0 otherwise. In order to more clearly observe how the ETS's distance attenuation evolves with respect to carbon reductions, this technique can eliminate the provinces that fall below the distance threshold from the spatial weight matrix.

10 $$ d = d_{\min } ,d_{\min } + \gamma ,d_{\min } + 2\gamma ,...,d_{\max } $$

11 $$ W_{i,j,d} = \left\{ {\begin{array}{*{20}c} {\frac{1}{{d_{i,j}^{2} }}, \, d_{i,j} \ge d} \\ {0, \, d_{i,j} < d} \\ \end{array} } \right.. $$

Utilizing the previously computed threshold distance spatial weight matrix, the Intensity Modified SDID Model could be expanded into the Intensity Modified SDID Model with Geographical Attenuation Process as Eq. (12) follows.

12 $$ \begin{gathered} CB_{i,t} = \gamma_{0} + \rho \sum\limits_{j = 1}^{N} {W_{i,t,d} CBEM_{i,t} } + \gamma_{1} TREND_{i,i} \times POST_{i,i} \times I + \gamma_{2} \sum\limits_{j = 1}^{N} {W_{i,t,d} } TREND_{i,i} \times POST_{i,i} \times I \hfill \\ \, + \phi X_{i,t} + \theta \sum\limits_{j = 1}^{N} {W_{i,t,d} X_{i,t} } + c_{i} + u_{t} + \varepsilon_{i,t} , \, \varepsilon_{i,t} \sim N(0,\sigma_{i,t}^{2} ), \hfill \\ \end{gathered} $$

### Descriptive statistics

**Figure 6:**
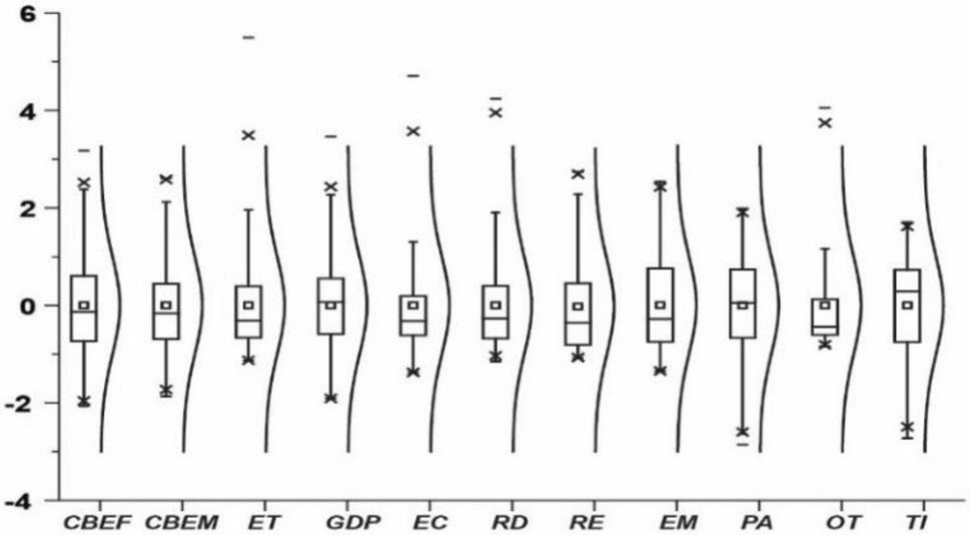
Descriptive statistics.

Figure 6 displays descriptive statistics for the employed variables using boxplots and distribution plots. The mean values are consistently centered around 0, with stable variances near 1, indicating stability achieved through mean standardization. The boxplots reject left- or right-biased distributions. Distribution plots confirm variables largely adhere to normal distribution characteristics, showcasing low volatility and high stability. These features make the chosen variables well-suited for model construction, eliminating the need for further preprocessing.

**Figure 7:**
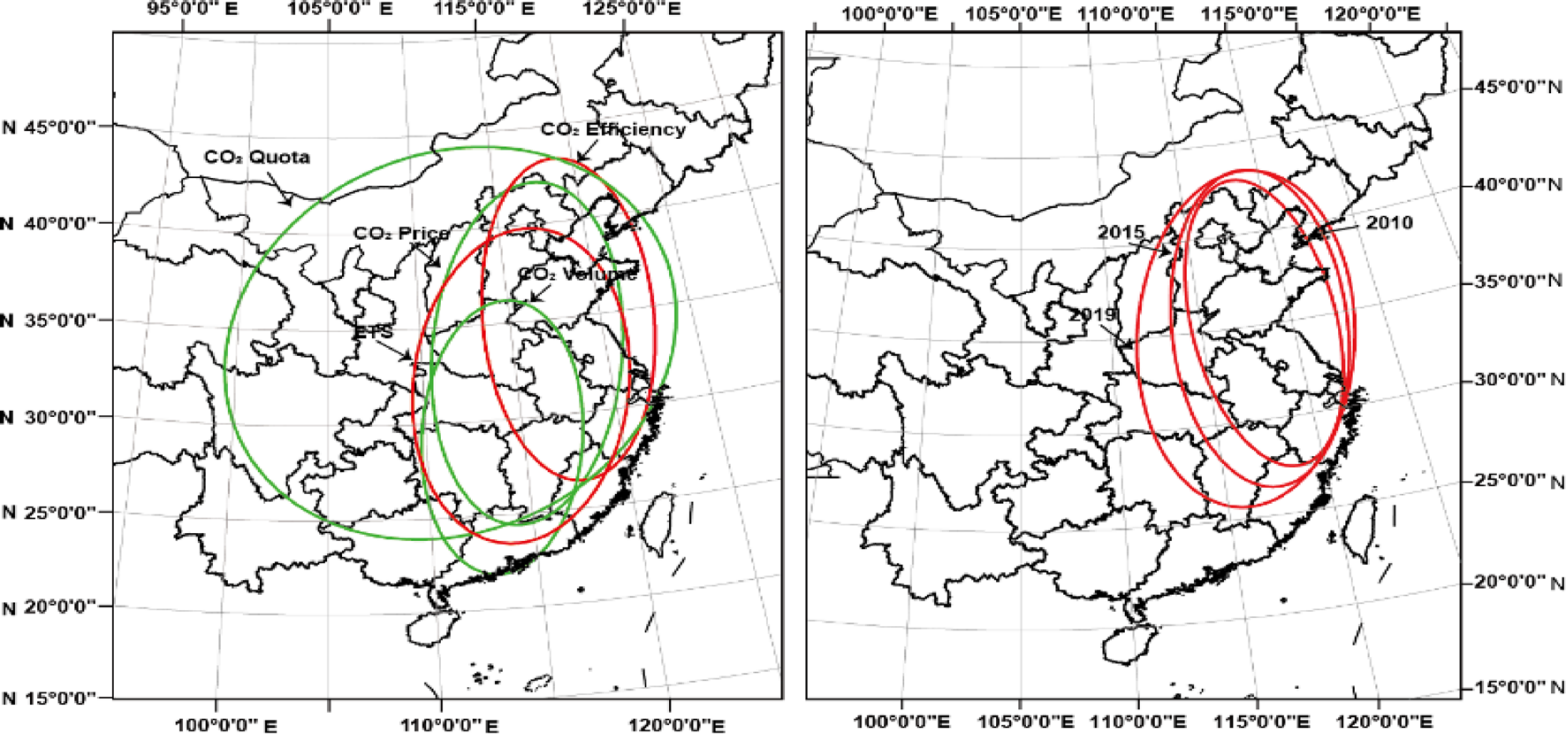
Spatial distribution and overlap among ETS and carbon reduction. Basemap data from the Resource and Environment Science and Data Center, Institute of Geographic Sciences and Natural Resources Research, Chinese Academy of Sciences (https://doi.org/10.12078/2023010103); Software version is ArcMap 10.6, URL is from https://www.esri.com/en-us/arcgis/products/indext.

In addition, spatial distribution and overlap analysis is conducted between carbon emissions and ETS. In Fig. 7(a), the impact of considering policy intensity on estimation is evident. The comparison of standard deviation ellipses for carbon dioxide emission efficiency and ETS (red ellipses) demonstrates a high correlation, around 80%, highlighting the crucial contribution of ETS in mitigating carbon emissions. However, comparing three elements within ETS (green ellipses), notable differences in ellipses are observed. In Fig. 7(b), the ellipses of carbon emissions exhibit a notable yearly fluctuation, highlighting the importance of the role of ETS. Prior to ETS, the standard deviation ellipse was oriented further north and east; post-ETS establishment, the ellipse shifted southwest, encompassing mainland China.

## Empirical results

### Influence mechanism of ETS on carbon reductions

Before empirical analysis, model validity is tested, and an appropriate baseline model is selected, with results presented in Table 2. Model 1 is a multi-stage DID model without fixed effects, Model 2 includes fixed effects, Model 3 showcases the SDM-DID model, Model 4 presents the SEM-DID model, Model 5 displays the SAR-DID model, and Model 6 is a DID model considering spatial terms for independent variables, dependent variables, and residuals. Multiple model comparisons confirm the robustness of ETS's carbon reduction effects, but the coefficients vary greatly among different model settings. Specifically, the coefficients of *Post* × *Trend* range from  − 0.09432 to − 0.0497 (models using *CBEM*) and 0.0311 to 0.0434 (models using *CBEF*), with significance levels stable between 5 and 10%. However, when spatial factors are considered, the influence intensity (i.e., coefficients of *W* × *Post* × *Trend*) decreases by around 12.3 ‰ to 59.40 ‰. This indicates that the neglect of spatial factors would lead to an overestimation, which is mainly due to the non-separating and ignoring of the influence intensity caused by neighboring regions. Additionally, likelihood ratio (LR) tests are also conducted, the results of which indicate LR statistics for both spatial residual and lag terms reject the null hypothesis. It proves the selection of Model 6 as the baseline model, which considers spatial terms in both independent variables, dependent variables, and residuals.

**Table 2:** Results of Baseline Estimation and LR tests.

Panel A: Models using Variable of Carbon Emission Intensity (*CBEM*)						
	(1)	(2)	(3)	(4)	(5)	(6)
*Post* × *Trend*	− 0.0497 (− 1.15)	− 0.0932 (− 1.74)*	− 0.0942 (− 3.86)***	− 0.0942 (− 3.86)***	− 0.0942 (− 3.86)***	− 0.0942 (− 3.86)***
*W* × *Post* × *Trend*			− 0.3486 (− 2.93)***	− 0.0348 (− 2.93)***	− 0.0348 (− 2.93)***	− 0.0348 (− 2.93)***
Control Variables	Yes	Yes	Yes	Yes	Yes	Yes
Spatial lag of Y	No	No	Yes	No	No	Yes
Spatial lag of X	No	No	Yes	No	Yes	Yes
Spatial lag of Error	No	No	No	Yes	No	Yes
Region fixed effect	No	Yes	Yes	Yes	Yes	Yes
R-sq	0.8104	0.8178	0.8183	0.8183	0.8183	0.8183
LM for error	193.916***					
Robust LM for error	123.852***					
LM for lag	74.791***					
Robust LM for lag	4.727**					
Panel B: models using variable of carbon emission efficiency (*CBEF*)						
	(7)	(8)	(9)	(10)	(11)	(12)
*Post* × *Trend*	0.0434 (1.97)**	0.0379 (1.18)	0.0311 (1.94)**	0.0311 (1.94)**	0.0311 (1.94)**	0.0311 (1.94)**
*W* × *Post* × *Trend*			0.0297 (1.73)*	0.0297 (1.73)*	0.0297 (1.73)*	0.0297 (1.73)*
Control Variables	Yes	Yes	Yes	Yes	Yes	Yes
Spatial lag of Y	No	No	Yes	No	No	Yes
Spatial lag of X	No	No	Yes	No	Yes	Yes
Spatial lag of Error	No	No	No	Yes	No	Yes
Region Fixed Effect	No	Yes	Yes	Yes	Yes	Yes
R-sq	0.5572	0.2718	0.3367	0.3367	0.3367	0.3367
LM for error	88.589***					
Robust LM for error	42.237***					
LM for lag	49.833***					
Robust LM for lag	3.481**					

The values in brackets represent standard errors. The symbol * indicates a p-value less than 0.1, ** indicates a p-value less than 0.05, and *** indicates a p-value less than 0.01. This notation will be used consistently throughout.

Intensity Modified SDID models are performed, to examine the influence mechanism process of ETS on carbon reductions, with results in Table 3 below. Table 3 presents findings on three elements, lower quotas, higher volume, and higher price, significantly reducing carbon emission intensity or promoting carbon emission efficiency. In Panel A, each 10 billion-ton reduction in carbon quotas results in a reduction (increase) of approximately 10.0% (2.57%) in carbon emission intensity (efficiency), with statistical significance at the 1% level across multiple models. In Panel B, each 10 thousand-ton growth in carbon volumes causes a decrease (increase) of approximately 2.73% (2.90‰) in carbon emission intensity (efficiency), with statistical significance at the 1% to 5% level across multiple models. In Panel C, each 10 RMB growth in carbon price causes a reduction (increase) of approximately 2.70% (1.30%) in carbon emission intensity (efficiency), with statistical significance at the 1% level across multiple models.

**Table 3:** Estimation results of Intensity Modified SDID models.

	DIRECT EFFECTS				INDIRECT EFFECTS			
	*CBEM*		*CBEF*		*CBEM*		*CBEF*	
	(1)	(2)	(3)	(4)	(5)	(6)	(7)	(8)
Panel A: Carbon Emission Quotas								
*Post* × *Trend* × *I*^*quota*^	2.02e − 03 (3.60)***	1.00e − 03 (3.49)***	− 4.74e − 04 (− 3.24)***	− 2.57e − 04 (− 1.60)	− 5.71e − 04 (− 2.82)***	− 2.27e − 04 (− 1.47)	0.0213 (2.19)**	3.06e − 03 (2.63)***
*ET*		0.0436 (3.00)***		0.0322 (3.98)***		0.0287 (2.79)***		0.1325 (2.48)**
*EC*		− 0.0202 (− 1.07)		0.0285 (2.88)***		0.0249 (2.53)**		− 0.0629 (− 1.01)
*RD*		0.1494 (3.64)***		0.0294 (1.65)*		0.0255 (1.56)		0.4480 (3.23)***
*GDP*		0.0004 (0.06)		− 0.0110 (− 2.56)**		− 0.0097 (− 2.22)**		0.0013 (0.06)
*RE*		0.0356 (2.78)***		− 0.0170 (− 2.39)**		− 0.0152 (− 1.94)*		0.1068 (2.57)***
*EM*		− 0.2633 (− 3.17)***		− 0.0286 (− 1.30)		− 0.0252 (− 1.22)		− 0.7940 (− 2.66)***
*PA*		0.1828 (7.69)***		0.0186 (1.40)		0.0166 (1.28)		0.5475 (5.23)***
*TO*		0.2256 (8.44)***		− 0.0269 (− 2.40)**		− 0.0236 (− 2.15)**		0.6864 (3.90)***
*TI*		0.0578 (1.55)		0.0148 (0.79)		0.0126 (0.73)		0.1695 (1.51)
rho	0.9467 (68.61)***	0.7902 (22.97)***	0.5718 (10.43)***	0.4891 (7.92)***	0.5718 (10.43)***	0.4891 (7.92)***	0.9467 (68.61)***	0.7902 (22.97)***
lgt_theta	− 3.2030 (− 22.51)***	− 3.0281 (− 19.68)***	− 1.6406 (− 10.10)***	− 1.5095 (− 8.68)***	− 1.6406 (− 10.10)***	− 1.5095 (− 8.68)***	− 3.2030 (− 22.51)***	− 3.0281 (− 19.68)***
sigma2_e	0.0088 (11.18)***	0.0056 (11.17)***	0.0029 (11.46)***	0.0026 (11.40)***	0.0029 (11.46)***	0.0026 (11.40)***	0.0088 (11.18)***	0.0056 (11.17)***
*R* − sq	0.6296	0.8210	0.0817	0.3352	0.0817	0.3352	0.6296	0.8210
Log − likelihood	142.9022	236.0815	382.8527	404.7420	382.8527	404.7420	142.9022	236.0815
Panel B: Carbon Trading Volumes								
	(9)	(10)	(11)	(12)	(13)	(14)	(15)	(16)
*Post* × *Trend* × *I*^*volume*^	− 7.00e − 06 (− 3.45)***	− 2.73e − 06 (− 2.62)***	1.21e − 06 (2.15)**	2.90e − 07 (0.50)	− 7.09e − 05 (− 2.13)**	− 8.36e − 06 (− 2.25)**	1.55e − 06 (2.00)**	2.65e − 07 (0.48)
*ET*		0.0410 (2.79)***		0.0325 (4.00)***		0.1256 (2.35)**		0.0297 (2.79)***
*EC*		− 0.0163 (− 0.86)		0.0267 (2.71)***		− 0.0514 (− 0.84)		0.0239 (2.41)**
*RD*		0.1278 (3.09)***		0.0365 (2.05)**		0.3853 (2.89)***		0.0325 (1.92)*
*GDP*		− 0.0032 (− 0.42)		− 0.0104 (− 2.38)**		− 0.0101 (− 0.41)		− 0.0093 (− 2.09)**
*RE*		0.0372 (2.83)***		− 0.0181 (− 2.51)**		0.1126 (2.58)***		− 0.0166 (− 2.01)**
*EM*		− 0.2381 (− 2.83)***		− 0.0311 (− 1.40)		− 0.7235 (− 2.44)**		− 0.0281 (− 1.31)
*PA*		0.1853 (7.74)***		0.0185 (1.38)		0.5597 (5.17)***		0.0168 (1.26)
*TO*		0.2376 (8.72)***		− 0.0318 (− 2.75)***		0.7291 (3.87)***		− 0.0286 (− 2.36)**
*TI*		0.0588 (1.51)		0.0157 (0.82)		0.1738 (1.46)		0.0137 (0.76)
rho	0.9438 (66.54)***	0.7918 (22.86)***	0.5898 (10.90)***	0.4956 (8.05)***	0.9431 (66.75)***	0.7918 (22.86)***	0.9438 (66.54)***	0.4956 (8.05)***
lgt_theta	− 3.1804 (− 22.36)***	− 3.0144 (− 19.34)***	− 1.6252 (− 9.98)***	− 1.5122 (− 8.69)***	− 3.2067 (− 22.55)***	− 3.0144 (− 19.34)***	− 3.1804 (− 22.36)***	− 1.5122 (− 8.69)***
sigma2_e	0.0090 (11.20)***	0.0057 (11.14)***	0.0030 (11.44)***	0.0026 (11.40)	0.0088 (11.20)***	0.0057 (11.14)***	0.0090 (11.20)***	0.0026 (11.40)
*R* − sq	0.6194	0.8256	0.0672	0.3227	0.5912	0.8256	0.6194	0.3227
Log − likelihood	142.0573	233.4450	379.7045	403.4566	144.1919	233.4450	142.0573	403.4566
Panel C: Carbon Trading Prices								
	(17)	(18)	(19)	(20)	(21)	(22)	(23)	(24)
*Post* × *Trend* × *I*^*price*^	− 0.0061 (− 3.84)***	− 0.0027 (− 3.38)***	0.0019 (4.48)***	0.0013 (2.74)***	− 0.0614 (− 2.30)**	− 0.0081 (− 2.83)***	0.0025 (3.36)***	0.0012 (2.19)***
*ET*		0.0474 (3.24)***		0.0301 (3.71)***		0.1395 (2.64)***		0.0287 (2.70)***
*EC*		− 0.0185 (− 0.99)		0.0295 (3.02)***		− 0.0557 (− 0.95)		0.0277 (2.62)***
*RD*		0.1444 (3.47)***		0.0218 (1.18)		0.4189 (3.19)***		0.0199 (1.12)
*GDP*		0.0007 (0.10)		− 0.0112 (− 2.63)***		0.0020 (0.09)		− 0.0105 (− 2.26)**
*RE*		0.0364 (2.80)***		− 0.0164 (− 2.30)**		0.1059 (2.54)**		− 0.0157 (− 1.89)*
*EM*		− 0.2862 (− 3.47)***		− 0.0262 (− 1.19)		− 0.8369 (− 2.86)***		− 0.0247 (− 1.12)
*PA*		0.1829 (7.69)***		0.0195 (1.48)		0.5310*** (5.29)		0.0186 (1.34)
*TO*		0.2243 (8.13)***		− 0.0223 (− 1.96)**		0.6624 (3.80)***		− 0.0208 (− 1.80)*
*TI*		0.0515 (1.36)		0.0155 (0.82)		0.1455 (1.32)		0.0141 (0.76)
rho	0.9431 (66.75)***	0.7843 (22.59)***	0.5916 (11.22)***	0.5074 (8.33)***	0.9431 (66.75)***	0.7843 (22.59)***	0.5916 (11.22)***	0.5074 (8.33)***
lgt_theta	− 3.2067 (− 22.55)***	− 3.0122 (− 19.63)***	− 1.6525 (− 10.19)***	− 1.5056 (− 8.67)***	− 3.2067 (− 22.55)***	− 3.0122 (− 19.63)***	− 1.6525 (− 10.19)***	− 1.5056 (− 8.67)***
sigma2_e	0.0088 (11.20)***	0.0056 (11.19)***	0.0028 (11.45)***	0.0026 (11.40)***	0.0088 (11.20)***	0.0056 (11.19)***	0.0028 (11.45)***	0.0026 (11.40)***
*R* − sq	0.5912	0.8433	0.0655	0.3494	0.5912	0.8433	0.0655	0.3494
Log − likelihood	144.1919	235.7131	387.3978	407.1751	144.1919	235.7131	387.3978	407.1751

### Spatiotemporal characteristics of ETS's carbon reduction influence mechanism

**Figure 8:**
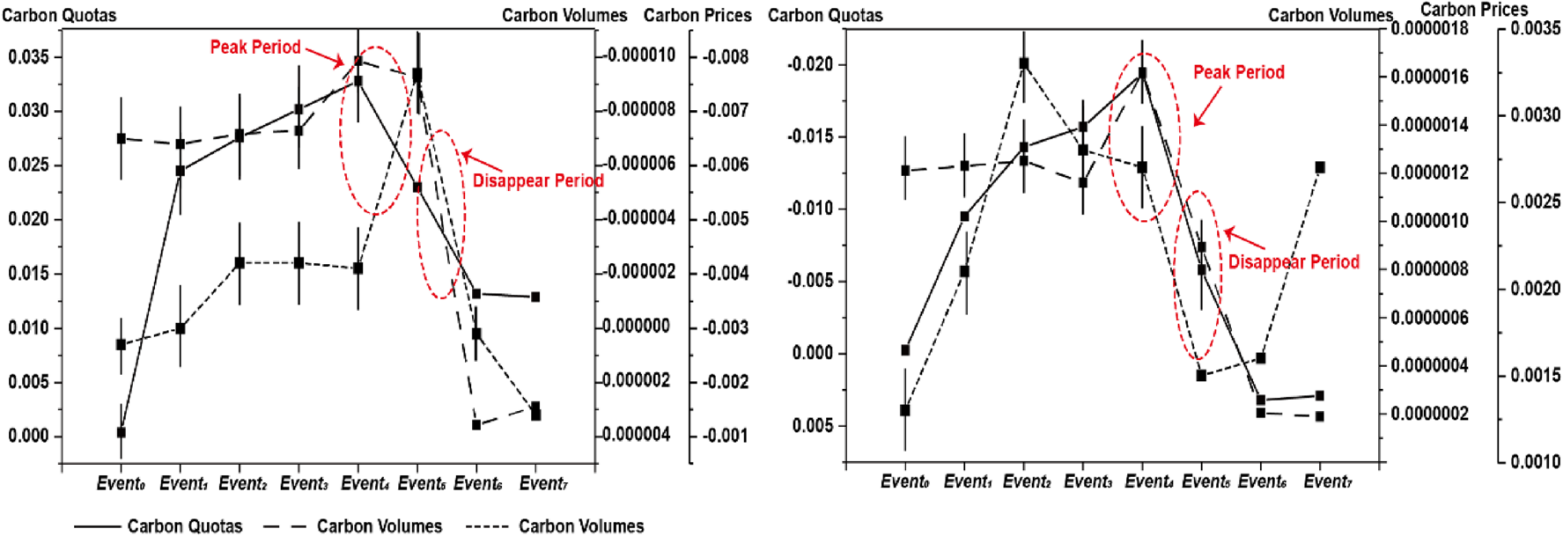
Dynamic changes.

To explore the changes in ETS’s effects over time, the Intensity Modified SDID Model with Dynamic Effects is performed with results in Table 4 and Fig. 8. Notably, there is a significant lag phenomenon in ETS's carbon reduction effects. Despite ETS proving effective since its establishment, its intensity gradually strengthened over time. For both models using *CBEM* and *CBEF*, the statistical indicators, coefficients, and *t*-statistics, of ETS's effects peak after a lag of 2 to 4 years (*Event*_+*2*_ to *Event*_+*4*_). and disappear around the 5th to 6th year (*Event*_+*5*_ to *Event*_+*6*_). Deeper exploration reveals that among the three elements of ETS, in comparison to quotas and volumes having the most substantial influence in the approximate 4th period (*Event*_+*4*_), the carbon reduction effects of prices advance by two periods and exhibit the strongest effect in the approximate second period (*Event*_+*2*_).

**Table 4:** Estimation results of Intensity Modified SDID Model with Dynamic Effects.

	DIRECT	INDIRECT		DIRECT	INDIRECT		DIRECT	INDIRECT
	(1)	(2)		(3)	(4)		(5)	(6)
Panel A: Models using Variable of Carbon Emission Intensity (*CBEM*)								
*Event*_*0*_ × *I*^*quota*^	0.0004 (1.85)*	0.0004 (1.56)	*Event*_*0*_ × *I*^*volume*^	-7.00e-06 (-3.45)***	-7.09e-05 (-2.13)**	*Event*_*0*_ × *I*^*price*^	-0.0027 (-3.38)***	-0.0081 (-2.83)***
*Event*_+*1*_ × *I*^*quota*^	0.0245 (3.18)***	0.0254 (2.30)**	*Event*_+*1*_ × *I*^*volume*^	-6.79e-06 (-3.47)***	-6.85e-05 (-2.14)**	*Event*_+*1*_ × *I*^*price*^	-0.0030 (-3.10)***	-0.0091 (-2.60)***
*Event*_+*2*_ × *I*^*quota*^	0.0276 (3.26)***	0.0288 (2.32)**	*Event*_+*2*_ × *I*^*volume*^	-7.17e-06 (-3.59)***	-7.28e-05 (-2.18)**	*Event*_+*2*_ × *I*^*price*^	-0.0042 (-3.48)***	-0.0132 (-2.73)***
*Event*_+*3*_ × *I*^*quota*^	0.0302 (3.18)***	0.0319 (2.28)**	*Event*_+*3*_ × *I*^*volume*^	-7.29e-06 (-3.66)***	-7.30e-05 (-2.23)**	*Event*_+*3*_ × *I*^*price*^	-0.0042 (-3.90)***	-0.0136 (-2.91)***
*Event*_+*4*_ × *I*^*quota*^	0.0328 (3.00)***	0.0351 (2.20)**	*Event*_+*4*_ × *I*^*volume*^	-9.86e-06 (-3.85)***	-9.86e-05 (-2.31)**	*Event*_+*4*_ × *I*^*price*^	-0.0041 (-4.30)***	-0.0136 (-3.08)***
*Event*_+*5*_ × *I*^*quota*^	0.0230 (1.24)	0.0239 (1.12)	*Event*_+*5*_ × *I*^*quota*^	-9.26e-06 (-3.17)***	-8.53e-05 (-2.17)**	*Event*_+*5*_ × *I*^*price*^	-0.0077 (-4.60)***	-0.0258 (-3.16)***
*Event*_+*6*_ × *I*^*quota*^	0.0132 (0.95)	0.0189 (1.00)	*Event*_+*6*_ × *I*^*volume*^	3.57e-06 (1.43)	3.85e-06 (1.35)	*Event*_+*6*_ × *I*^*price*^	-00,029 (-2.40)**	-00,085 (-2.16)**
*Event*_+*7*_ × *I*^*quota*^	0.0129 (0.83)	0.0175 (0.79)	*Event*_+*7*_ × *I*^*volume*^	2.89e-06 (1.39)	2.67e-06 (1.31)	*Event*_+*7*_ × *I*^*price*^	-0.0014 (1.45)	-0.0067 (1.34)
Panel B: Models using Variable of Carbon Emission Efficiency (*CBEF*)								
	(7)	(8)		(9)	(10)		(11)	(12)
*Event*_*0*_ × *I*^*quota*^	-2.57e-4 (-1.60)	-2.27e-4 (-1.47)	*Event*_*0*_ × *I*^*volume*^	1.21e-06 (2.15)**	1.55e-06 (2.00)**	*Event*_*0*_ × *I*^*price*^	0.0013 (2.74)***	0.0012 (2.19)**
*Event*_+*1*_ × *I*^*quota*^	-0.0095 (-1.47)	-0.0086 (-1.35)	*Event*_+*1*_ × *I*^*volume*^	1.23e-06 (2.25)**	1.56e-06 (2.09)**	*Event*_+*1*_ × *I*^*price*^	0.0021 (3.96)***	0.0019 (2.83)***
*Event*_+*2*_ × *I*^*quota*^	-0.0143 (-2.18)**	-0.0126 (-1.93)**	*Event*_+*2*_ × *I*^*volume*^	1.25e-06 (2.26)**	1.57e-06 (2.11)**	*Event*_+*2*_ × *I*^*price*^	0.0033 (5.45)***	0.0028 (3.42)***
*Event*_+*3*_ × *I*^*quota*^	-0.0157 (-2.52)**	-0.0135 (-2.19)**	*Event*_+*3*_ × *I*^*volume*^	1.16e-06 (2.05)*	1.46e-06 (1.94)*	*Event*_+*3*_ × *I*^*price*^	0.0028 (5.07)***	0.0023 (3.37)***
*Event*_+*4*_ × *I*^*quota*^	-0.0194 (-3.20)***	-0.0161 (-2.63)***	*Event*_+*4*_ × *I*^*volume*^	1.62e-06 (2.23)**	2.00e-06 (2.12)**	*Event*_+*4*_ × *I*^*price*^	0.0027 (5.17)***	0.0022 (3.41)***
*Event*_+*5*_ × *I*^*quota*^	-0.0058 (-0.62)	-0.0049 (-0.58)	*Event*_+*5*_ × *I*^*quota*^	8.93e-07 (0.93)	1.13e-06 (0.88)	*Event*_+*5*_ × *I*^*price*^	0.0015 (1.64)	0.0013 (1.55)
*Event*_+*6*_ × *I*^*quota*^	0.0032 (0.62)	0.0089 (0.95)	*Event*_+*6*_ × *I*^*volume*^	2.05e-07 (1.73)	2.67e-07 (1.62)	*Event*_+*6*_ × *I*^*price*^	0.0016 (1.26)	0.0008 (1.02)
*Event*_+*7*_ × *I*^*quota*^	0.0029 (0.93)	0.0075 (0.56)	*Event*_+*7*_ × *I*^*volume*^	1.89e-07 (1.04)	1.63e-07 (1.21)	*Event*_+*7*_ × *I*^*price*^	0.0027 (1.35)	0.0010 (1.12)

**Figure 9:**
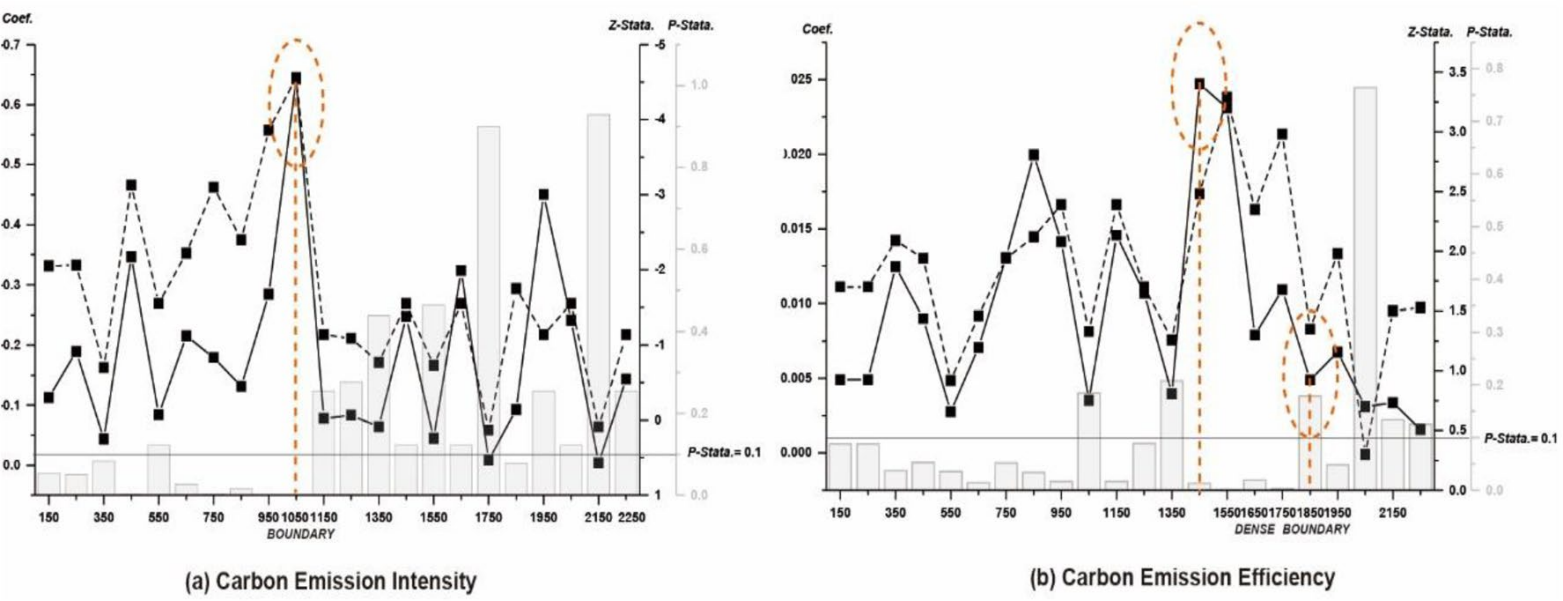
Geographical Attenuation Process.

Based on the attenuation law, the spatial effect attenuates with the deepening of the geographical distance among provinces, including boundaries and peaks. To capture this attenuation process, Intensity modified SDID Model with the geographical attenuation process is performed, with results in Fig. 9. Regarding the attenuation boundary, the carbon reduction effects exist within about 1500km, the spillover effect would disappear exceeding 1500km. For the carbon intensity model, when exceeding 1000km, coefficients and significant levels drop sharply, indicating the insignificance of spillover effects. Similarly, or the carbon efficiency model, when exceeding 1700km, coefficients and significant levels decrease sharply. Regarding influence peaks areas, the carbon reduction effects reach their strongest in areas around 1,000km, forming peaks areas. For the carbon intensity model, when exceeding 1000km, coefficients and significant levels reach the peak value, indicating the peak areas of spillover effects. Similarly, for the carbon efficiency model, when exceeding 1500km, various statistical values reach the peak value.

### Heterogeneities of ETS's carbon reduction effects

#### Pollutants heterogeneity

**Figure 10:**
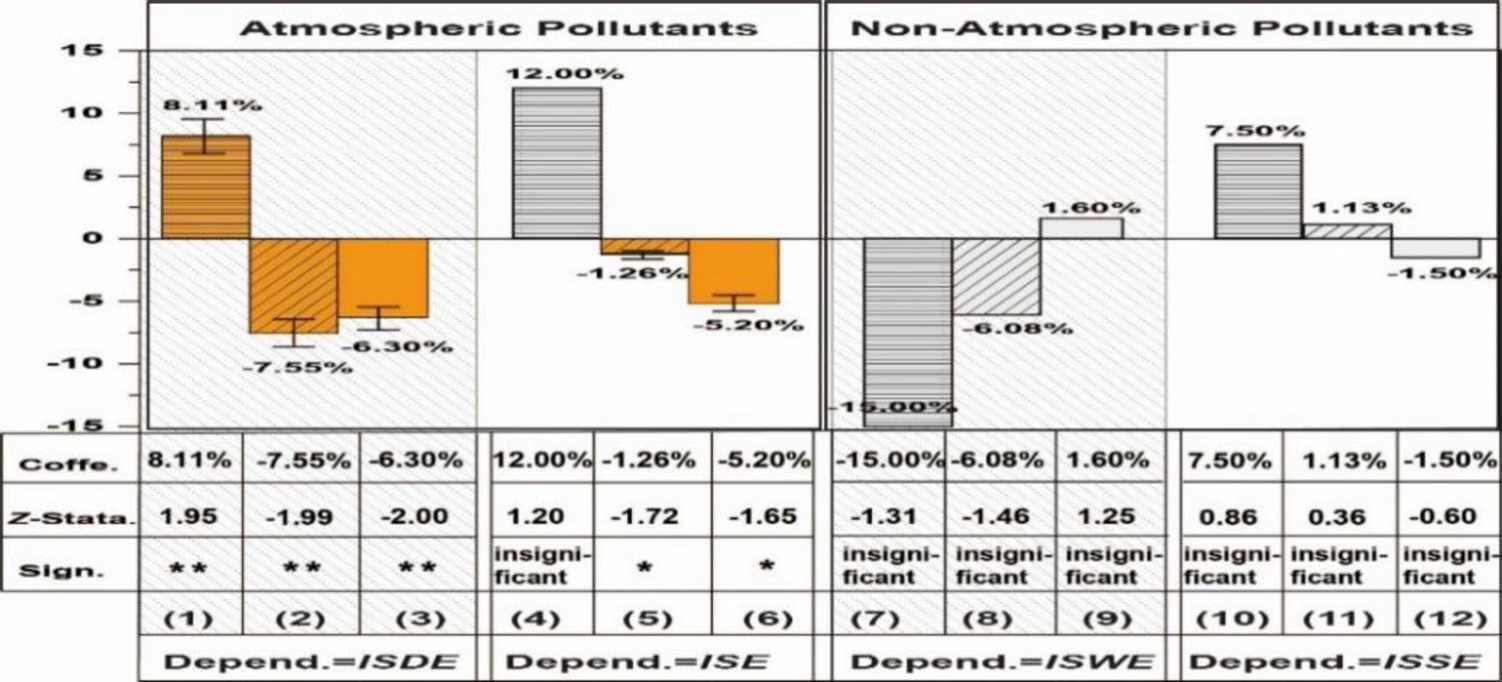
Comparisons of heterogenous pollutants’ carbon reduction effects.

Considering the same source of carbon dioxide and other pollutants, ETS has the potential to not only decrease carbon emissions but also have a positive impact on the emissions of other pollutants through synergistic effects. Therefore, heterogeneities analysis is performed firstly from the aspect of pollutants. This study adopts industrial emissions of sulfur dioxide, smoke (dust), wastewater, and solid waste as proxy indexes, with results in Table 5 and Fig. 10.

**Table 5:** Results of pollutants heterogeneity.

	Industrial sulfur dioxide emission (*ISDE*)			Industrial smoke (dust) emission (*ISE*)			Industrial wastewater emission (*IWWE*)			Industrial solid waste emission* (ISWE*)		
	(1)	(2)	(3)	(4)	(5)	(6)	(7)	(8)	(9)	(10)	(11)	(12)
*Post* × *Trend* × *I*^*quota*^	− 0.0063 (− 2.00)**			− 0.0052 (− 1.65)*			0.0016 (1.25)			− 0.0015 (− 0.60)		
*Post* × *Trend* × *I*^*volume*^		− 7.55e − 06 (− 1.99)**			− 1.26e − 06 (− 1.72)*			− 6.08e − 06 (− 1.46)			1.13e − 06 (0.36)	
*Post* × *Trend* × *I*^*price*^			8.11e − 04 (1.95)**			0.0012 (1.20)			− 0.0015 (− 1.31)			7.50e − 04 (0.86)
Control variables	Yes	Yes	Yes	Yes	Yes	Yes	Yes	Yes	Yes	Yes	Yes	Yes
*rho*	0.8061 (19.50)***	0.8007 (19.19)***	0.8054 (19.51)***	0.7724 (17.31)***	0.7711 (17.18)***	0.7760 (17.51)***	0.2094 (2.31)**	0.2036 (2.25)**	0.2033 (2.24)**	0.1714 (1.73) *	0.1796 (1.82)*	0.1731 (1.75)*
lgt_theta	− 0.9516 (− 3.87)***	− 0.8971 (− 3.74)***	− 0.9993 (− 4.10)***	− 1.0927 (− 4.79)***	− 1.1132 (− 4.88)***	− 1.099 (− 4.88)***	− 1.2272 (− 5.87)***	− 1.1982 (− 5.78)***	− 1.2505 (− 6.03)***	− 1.7969 (− 10.49)***	− 1.8107 (− 10.61)***	− 1.8024 (− 10.55)***
sigma2_e	0.1474 (10.86)***	0.1473 (10.92)***	0.1448 (10.89)***	0.1473 (10.91)***	0.1476 (10.90)***	0.1462 (10.93)***	0.1705 (11.35)***	0.1711 (11.37)***	0.1691 (11.35)***	0.0888 (11.52)***	0.0887 (11.52)***	0.0888 (11.52)***
*R*-sq	0.5343	0.5942	0.5353	0.3760	0.3537	0.3733	0.5954	0.6148	0.5832	0.4494	0.4319	0.4431
Log-likelihood	− 200.6512	− 198.9549	− 198.9481	− 201.6149	− 201.6149	− 200.3071	− 206.0680	− 205.8651	− 205.3131	− 121.8153	− 122.1185	− 122.0055

ETS also has synergistic effects with atmospheric pollution reduction, including industrial emissions of sulfur dioxide and smoke (dust), but are insignificant to industrial emissions of wastewater and solid waste. Specifically, in models considering industrial *ISDE*, each increasing unit of ETS's three elements would lead to around 6.30% to 8.11% reduction of carbon emission. In models considering *ISE*, each increasing unit of carbon trading price and volume would lead to around 1.26% to 5.20% reduction of carbon emission. However, the regression coefficients for industrial emissions of solid waste and wastewater are not statistically significant.

#### Regional heterogeneity

**Figure 11:**
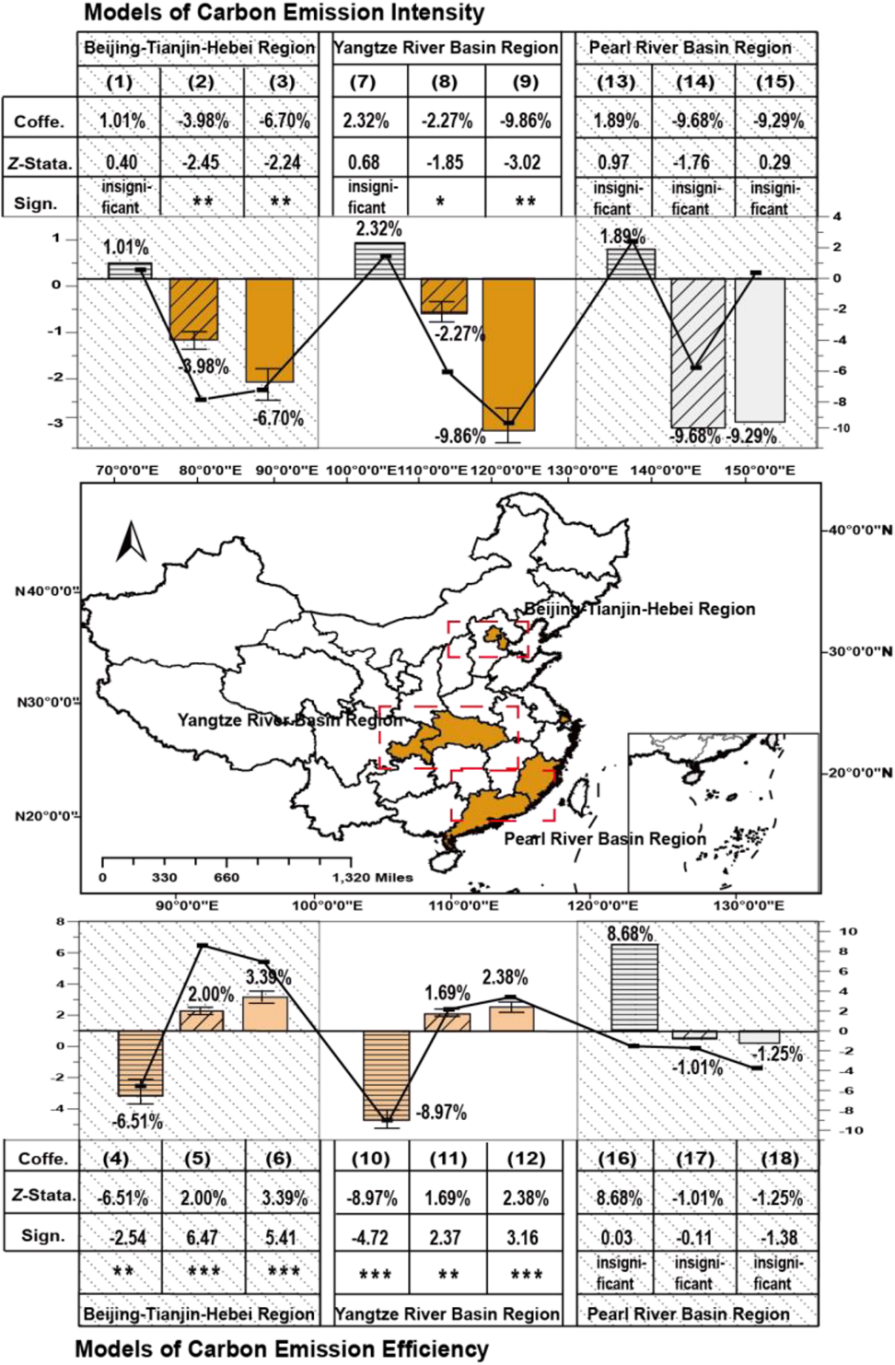
Comparisons of heterogenous regions’ carbon reduction effects. Basemap data from the Resource and Environment Science and Data Center, Institute of Geographic Sciences and Natural Resources Research, Chinese Academy of Sciences (https://doi.org/10.12078/2023010103); Software version is ArcMap 10.6, URL is from https://www.esri.com/en-us/arcgis/products/indext.

Considering the geographical clustering characteristics of pilot markets, the heterogeneities analysis is also performed from geographical clustering, with results in Table 6 and Fig. 11. China's eight pilot markets are mainly concentrated in three regions, including the Beijing-Tianjin-Hebei region in northern China, the Yangtze River Delta region in China's economic center, and the Pearl River Delta region in China's foreign trade center.

**Table 6:** Results of regional heterogeneity.

	Carbon Emission Intensity (*CBEM*)			Carbon Emission Efficiency (*CBEF*)		
	(1)	(2)	(3)	(4)	(5)	(6)
Panel A: Beijing-Tianjin-Hebei						
*Post* × *Trend* × *I*^*quota*^	1.01e − 03 (0.40)			− 6.51e − 04 (− 2.54)**		
*Post* × *Trend* × *I*^*volume*^		− 3.98e − 05 (− 2.45)**			2.00e − 05 (6.47)***	
*Post* × *Trend* × *I*^*price*^			− 6.70e − 03 (− 2.24)**			3.39e − 03 (5.41)***
*Post* × *Trend* × *I*^*quota*^	3.11e − 03 (0.12)			− 8.10e − 05 (− 0.99)		
*Post* × *Trend* × *I*^*volume*^		− 1.92e − 04 (− 1.57)			3.47e − 06 (1.51)	
*Post* × *Trend* × *I*^*price*^			− 0.0316 (− 1.52)			6.91e − 04 (1.60)
Contr. variables	yes	yes	yes	Yes	yes	yes
R-sq	0.6320	0.6263	0.6400	0.2043	0.3090	0.0753
Log-likelihood	39.6720	40.7789	37.1066	139.4094	144.0423	129.5856
Panel B: Yangtze River Delta						
	(7)	(8)	(9)	(10)	(11)	(12)
*Post* × *Trend* × *I*^*quota*^	2.32e − 03 (0.68)			− 8.97e − 05 (− 4.72)***		
*Post* × *Trend* × *I*^*volume*^		− 2.27e − 06 (− 1.85)*			1.69e − 06 (2.37)**	
*Post* × *Trend* × *I*^*price*^			− 9.86e − 03 (− 3.02)**			2.38e − 03 (3.16)***
*Post* × *Trend* × *I*^*quota*^	0.0131 (0.32)			− 1.66e − 03 (− 3.39)***		
*Post* × *Trend* × *I*^*volume*^		− 9.08e − 06 (− 0.75)			3.28e − 06 (1.97)**	
*Post* × *Trend* × *I*^*price*^			− 0.0453 (− 2.16)**			4.44e − 03 (2.49)**
Contr. variables	yes	yes	yes	Yes	yes	yes
R-sq	0.6557	0.4268	0.5946	0.1284	0.0738	0.1406
Log-likelihood	45.3600	40.4025	42.8264	205.1820	203.0449	210.1138
Panel C: Pearl River Delta						
	(13)	(14)	(15)	(16)	(17)	(18)
*Post* × *Trend* × *I*^*quota*^	1.89e − 03 (0.97)			8.68e − 06 (0.03)		
*Post* × *Trend* × *I*^*volume*^		− 9.68e − 06 (− 1.76)			− 1.01e − 07 (− 0.11)	
*Post* × *Trend* × *I*^*price*^			− 9.29e − 04 (0.29)			− 1.25e − 03 (− 1.38)
*Post* × *Trend* × *I*^*quota*^	0.0042 (0.62)			3.51e − 05 (0.13)		
*Post* × *Trend* × *I*^*volume*^		− 2.47e − 04 (− 1.30)			− 1.11e − 07 (− 0.17)	
*Post* × *Trend* × *I*^*price*^			− 1.79e − 03 (− 0.23)			− 8.37e − 04 (− 1.09)
Contr. variables	Yes	Yes	Yes	Yes	Yes	Yes
R-sq	0.8382	0.6931	0.8676	0.1171	0.1211	0.1094
Log-likelihood	13.5451	16.7883	14.5717	59.9117	58.9698	58.9643

In Table 6, carbon reduction effects in the Beijing-Tianjin-Hebei and Yangtze River Delta regions surpass the national average by 2 to 5 times and 3 to 7 times, respectively. However, coefficients in the Pearl River Delta region are not statistically significant. Specifically, each unit reduction in carbon quotas and each unit growth in carbon volumes and prices cause a 1.01% to 6.70% (6.51‰ to 3.39%) and 2.27‰ to 9.86% (1.69‰ to 2.38%) increase in carbon emission intensity (efficiency) in the Beijing-Tianjin-Hebei and Yangtze River Delta regions, respectively.

#### Placebo tests

**Figure 12:**
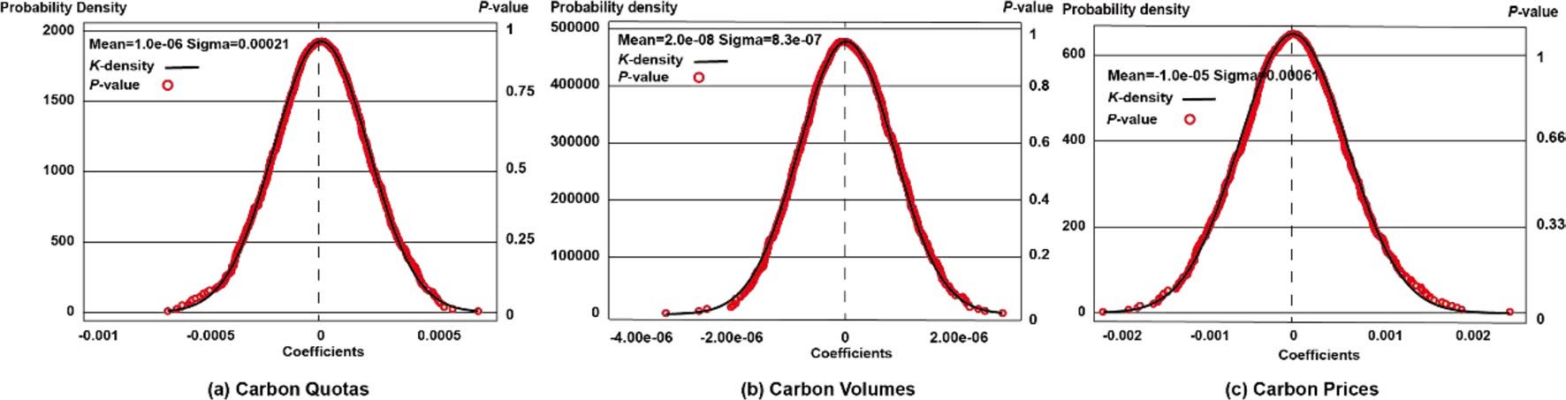
Results of placebo tests.

Placebo testing involves randomly selecting 5% of cross-sections as ETS pilot markets and randomly determining pilot years to assess whether observed carbon emission decreases are influenced by unobservable factors. Equations (6) to (8) were then iterated using simulated information, and the resulting distribution was visualized in Fig. 12(a), (b), and (c).

In the placebo tests, the estimates for carbon quotas, volumes, and prices are close to zero, specifically 1.08e-6, 2.0e-8, and -1.0e-6, respectively. This rejection of the hypothesis that other factors influence carbon emission reduction strongly supports the identification of our DID model. It affirms that constraining on carbon quotas, as well as expanding on carbon volumes and prices of ETS indeed lead to carbon reduction, forming the primary conclusions of the study.

## Discussions

### Influence mechanism of ETS

The influence mechanism process of ETS's carbon reductions could be summarized as the characteristic of "Dual circulation", internal and external mechanism paths overlap to form a comprehensive mechanism process. External mechanism pathways refer to the direct role of three elements of ETS on carbon reductions. The contracting of carbon emission quotas, as well as the increase in carbon trading prices and volumes, would directly result in the reduction of carbon emissions. From the statistical results, external mechanism pathways (i.e., direct roles of EST on carbon reductions) could also be proved. For each unit reduction in carbon quotas (10 billion-ton), or for each unit increase in carbon trading volume (10 thousand-ton) or carbon trading price (10 RMB), carbon emission intensity (efficiency) could be reduced (increased) by around 2.70% to 10.0% (2.90‰to 2.57%), all statistical indicators maintain significance levels of at least 5%. Even though this study marks the initial examining of the mechanism of ETS, a limited number of articles explored carbon reductions of carbon prices. For example, the counterfactually estimating results in the current study proved the carbon reductions of carbon prices when it cooperated with technological innovations^31^. Additionally, it is clear from the study that there is a constant and strong relationship between the amount of transactions in ETS pilots and the amount of CO_2_ that is reduced^10^.

The internal pathways refer to the interconnectedness among the three elements within ETS, collaborating with the external mechanism path to generate enhanced carbon reduction effects. The tightening of carbon emission quotas stimulates carbon trading volumes, thereby pushing up carbon prices^16^. Through interactions the reduction effects in internal and external pathways synergize, creating a substantially elevated platform for carbon reduction effects. From the statistical results of dynamic effects, internal mechanism paths could also be proved. Compared to quotas and volume with the highest influence in the approximate fourth period (*Event*_+*4*_), the carbon reduction effects of price advance by two periods and have the strongest effect in the approximate second period (*Event*_+*2*_). This emphasizes three elements play roles in sequence, the carbon reduction effects thereby are superimposed.

The "Dual Carbon Target" is a commitment declared by the Chinese government, aiming for carbon peaking by 2030 and carbon neutrality by 2060, however, it could not be achieved if maintaining the current performance of ETS markets. Based on empirical results, reasonable levels of quotas, volumes, and prices are proposed in this article for contributing to the "Dual Carbon Target". Estimates indicate that a nationwide reduction of approximately 20% to 25% in carbon emissions is necessary by 2030 to fulfill the Chinese government's "Dual Carbon Target"^10^.

Estimates suggest that carbon quotas need to be constrained to decrease by approximately 20 billion tons per year to achieve the "Dual Carbon Target." Achieving this goal remains challenging with the current quota management system. However, forecasting indicates that, beyond the existing power industry, additional high-carbon emission industries may be brought into the quotas management domain, expanding the management space for constraining carbon quotas in the future^32^.

Estimates indicate that carbon trading volumes are supposed to increase to around 150 thousand tons per year, showing an improvement of approximately 80 thousand tons from the current level. However, specific goals for carbon trading volumes vary across different pilot markets due to their distinct scales. Notably, the Hubei market requires the most significant increase, with an approximate rise of 60 million tons per year^9^. For meeting the "Dual Carbon Target", estimates propose an increase in carbon trading prices by about 100 RMB (14 USD) per ton, reaching approximately 150 to 200 RMB (21 to 28 USD) per ton. Significant room for improvement exists across all pilot markets, with prices ideally rising to approximately 180, 150, 160, 130, 110, 140, 130, and 110 RMB per ton in the markets of Beijing, Shanghai, Guangdong, Tianjin, Shenzhen, Hubei, Chongqing, and Fujian, respectively. Additionally, if China's carbon trading price can reach the level of Europe's ETS markets at 80 euros per ton, nationwide annual carbon emissions could be reduced by about 100%, thereby achieving China's "Carbon Peak Target" by 2060^10,33^. While this study is the first to compute and propose reasonable levels of quotas and volumes, limited articles have addressed the issue of reasonable pricing in carbon trading. Studies by^33,34^ have computed prices at levels similar to those calculated in this article.

### Spatiotemporal characteristics

Based on extensions of the Intensity Modified SDID Model (with dynamics and geographical attenuation process), spatiotemporal features of the influence mechanism are discussed. Concerning dynamic effects, following the establishment of the ETS, for both carbon intensity and efficiency, coefficients and significances peak in the 4th period (*Event*_+*4*_) and become insignificant in the 5th period (*Event*_+*5*_). This aligns with reality, reflecting the cautious approach of pilot programs, which originally gave businesses small quotas and led to low volumes and prices for carbon trading^10^. In the early stages, levels of carbon trading volumes and prices were only half of the current status, leading to low-impact intensities and significances^33,34^. However, by 2020 (*Event*_+*4*_), ETS markets gradually stabilized, reaching relatively high levels of influence intensities and significances.

Dynamic effects vary among the three elements of the ETS. While quotas and volumes exhibit the highest influence in the approximate 4th period (*Event*_+*4*_), the carbon reduction effects of price show advancement by two periods, exerting the strongest impact in the approximate second period (*Event*_+*2*_). This aligns with the theory of "dual circulation" proposed in this study, emphasizing that carbon prices, as the final link in the mechanism process, directly engage with the markets and enterprises, improving emission reduction or efficiency through cost pressure^10^.

Additionally, the spatial attenuation process of ETS's carbon emission reduction effects is clarified, including attenuation boundaries and influence peak areas. Regarding the attenuation boundaries, carbon reduction effects exist within about 1500km. For influence peak areas, the carbon reduction effects reach their strongest in areas around 1,000km, forming influence peak areas.

The current literature could provide proof and explanation of the spatial attenuation process^13,19^. Theories of regional protectionism, local preference, and non-standardized information all lend credence to the attenuation phenomenon. Non-standardized information, prone to loss during long-distance transmission, establishes regional attenuation processes^35^. Financial institutions, to minimize costs, prefer local collaborations, influencing spatial spillover effects. Government intervention and local protectionism contribute to regional boundaries in ETS.

### Heterogeneities

The ETS’ carbon reduction effects are influenced by pollutants and regional heterogeneities. Regarding the heterogeneity of pollutant types, ETS also has synergistic effects with atmospheric pollution reduction, including industrial emissions of sulfur dioxide and smoke (dust), but are insignificant to industrial emissions of wastewater and solid waste. Coal combustion, as the same source for gas pollutants emissions, mainly contributes to ETS's synergistic effects. China's coal use is the primary contributor to carbon emissions in the energy production cycle, meanwhile, it also emits a large amount of sulfur dioxide and smoke (dust)^10,36^. Therefore, the ETS mechanism can indirectly reduce the use of coal to reduce sulfur dioxide and smoke (dust) emissions^37^. Conversely, the compositions of solid and liquid pollutants are much more complex, where coal-fired plants contribute at a comparatively low rate. For example in Beijing city, in 2018, in the sources of solid pollutants, only less than 10% are from coal-fired sources, resulting in the insignificance of ETS's effects on industrial emissions of solid waste and wastewater^10^. In short, industrial sulfur dioxide and smoke (dust) emissions have been significantly reduced, primarily as a result of ETS's synergistic effects, both of which are mainly emitted from coal combustion. Atmospheric pollutants' systemic effects are also confirmed in plenty of articles. For example, the results of the study show that with the help of ETS, atmospheric pollutants like CO2 and SO2 were able to reduce their emissions in tandem, with the latter having the most noticeable synergistic effect^36^. Similarly, the study indicates that in the pilot locations, the ETS can reduce haze pollution and help reduce CO2 emissions, creating a win–win situation^37^.

For regional heterogeneities, carbon reduction effects in both regions of Beijing-Tianjin-Hebei and Yangtze River Delta are higher than the national average 2 to 5 and 3 to 7 times, respectively, while coefficients in the Pearl River Delta region are not statistically significant. Estimations results of regional heterogeneities are very intuitive. On the one hand, the pilot markets in the regions of Beijing-Tianjin-Hebei and the Yangtze River Delta have the smallest ETS size, which determines the significant expansion effects, while the Pearl River Delta region is with the lowest carbon trading price, thus, leading to insignificant reduction effects on carbon emissions^10,38^. On the other hand, regions of Beijing-Tianjin-Hebei and the Yangtze River Delta, as the pollical and economic centers of China, where carbon reduction technologies are much more advanced. Therefore, under the same conditions, such regions could promote greater carbon reduction effects by leveraging energy-saving infrastructure and sustainable technologies^39^.

## Research conclusions and implications

### Research conclusions

Traditional SDID models struggle to capture changes in policy intensity, leading to a lack of understanding of policy effectiveness in prior research on ETS. In response, this study introduces the newly constructed Intensity Modified SDID Model to delve into the influence mechanism of ETS's carbon reductions. Furthermore, through extensions of the Intensity Modified SDID Model, with dynamics effects and geographical attenuation processes, the study explores the spatiotemporal features of the influence mechanism, along with the heterogeneity of ETS's carbon reduction effects are also delved into. Several main findings could be obtained as follows.

Firstly, "dual-circulation" is a central feature in the ETS’s carbon reduction influence process. In the fundamental external pathways, ETS directly contributes to carbon reductions, with each unit change in quotas, volumes, and prices influencing carbon reduction by approximately 2.70% to 10.0%. Meanwhile, through internal connections and pathways, the reduction effects are continuously strengthened, and the increase in demand resulting from quotas' constraints pushes up carbon volumes and prices. With the combination of the influence of internal and external pathways, a more intensified influence mechanism is achieved.

Secondly, for achieving China's government's "Dual Carbon Target", reasonable setting levels of ETS’s quotas, volumes, and prices are estimated and proposed. Estimates in this article propose constraining the nationwide carbon quotas by around 20 billion tons per year, increasing the carbon trading volumes to about a hundred thousand tons per year, and elevating the carbon trading prices by about 100 RMB (14 USD) per ton to a range of approximately 150 to 200 RMB (21 to 28 USD) per ton.

Thirdly, ETS's carbon reduction effects are confirmed with temporal and spatial features, depending on time and region varieties. Temporally, ETS's carbon reduction effects peak in the 4th period (*Event*_+*4*_) but diminish in the 5th period (*Event*_+*5*_). Spatially, the effects peak maximum in areas distancing around 1000km but disappear beyond 1500km.

Fourthly, ETS also has synergistic effects with atmospheric pollution reduction, including industrial emissions of sulfur dioxide and smoke (dust), but are insignificant to industrial emissions of wastewater and solid waste. each increasing unit of ETS's three elements would lead to 1.26% to 8.11% reduction of atmospheric pollutants emission, but the regression coefficients for industrial emissions of solid waste and wastewater are not statistically significant.

This article introduces several contributions and innovative aspects. Firstly, this study constructs a novel Intensity Modified SDID Model, incorporating policy intensity as an indicator variable into traditional SDID models. This novel model addresses the limitations of traditional DID methods, which overlook the dynamic nature of ETS policies after establishment. Consequently, the Intensity Modified SDID Model overcomes simulation estimation bias, accurately capturing the evolving situation of the ETS market and providing a precise assessment of ETS's true emission reduction effects. Additionally, a mechanism analysis of ETS for reducing carbon emissions can be accomplished with the inclusion of policy intensity.

Secondly, leveraging the newly devised Intensity Modified SDID Model, this article pioneers an investigation into the carbon reduction impact mechanism of ETS. Unlike past approaches that merely compared carbon emission gaps pre- and post-ETS implementation, this study employs a more nuanced examination, delving into the intricate mechanisms, processes, and pathways of emission reduction. The Intensity Modified SDID Model reveals "dual-circulation" features in the carbon reduction impact mechanism of ETS. The external pathway exerts a direct effect, while the emission reduction effect is further bolstered through internal connections and pathways. The amalgamation of internal and external influences results in a more robust impact mechanism.

Thirdly, through extensions on the Intensity Modified SDID Model with dynamic effects and geographical attenuation processes, the study explores the spatiotemporal variation characteristics of ETS’s carbon reduction effects. In essence, policy effects exhibit variations over time and geography, and ETS’s carbon effects encompass both temporal and spatial features. Traditional DID models, lacking consideration for policy intensity, fall short in capturing temporal and spatial changes. The introduction of dynamic effects and geographical attenuation processes allows for the study of the trend of ETS effects over time and geography, representing an innovation compared with existing literature.

### Policy implications

Specific policy implications result from this study's conclusions. The central government is recommended to consider expanding the ETS market and setting reasonable levels. For one aspect, the establishment of a national ETS market is suggested, encompassing more regions and industries within the carbon emission management system. This expansion is crucial given the proven significant carbon reduction effects, creating both direct and synergistic effects from the interplay of the three ETS elements. Currently limited to pilot provinces, municipalities, and the power industry, there's an urgent need to extend the market nationwide, encompassing a broader spectrum of industries.

For another, it is recommended to contract carbon quotas, as well as increase carbon volumes and prices. More reasonable ETS levels can not only achieve stronger emission reduction effects but also contribute to China’s "Dual Carbon Target". Based on estimations in this study, the current performances of the ETS market would hold back the achievement of the "Dual Carbon Target". Estimates in this article propose constraining the nationwide carbon quotas by around 20 billion tons per year, increasing the carbon trading volumes to 150 thousand tons per year, and elevating the carbon trading prices by about 100 RMB (14 USD) per ton to a range of approximately 150 to 200 RMB (21 to 28 USD) per ton.

For local governments, the findings in this article highlight two key policy implications considerations. On the one hand, environmental authorities in the Pearl River Delta region should closely monitor the efficacy of the ETS pilot market. While most ETS markets exhibit success in achieving emission reduction targets, the effectiveness of the ETS pilot market in the Pearl River region appears less pronounced. Hence, addressing the inefficiencies in carbon emission reduction in the Pearl River Delta demands special attention.

On the other hand, regional environmental authorities should establish inter-provincial cooperation models. Using data on geographical attenuation boundaries and dense areas, authorities can identify suitable collaborative partners within a range of about 1,500km and in geographically dense areas. This streamlined process improves the efficiency of inter-provincial cooperation.

## Data Availability

The datasets used and/or analyzed during the current study are available from the corresponding author upon reasonable request.
